# Exceptionally Long Covalent CC Bonds—A Local Vibrational Mode Study

**DOI:** 10.3390/molecules26040950

**Published:** 2021-02-11

**Authors:** Alexis Antoinette Ann Delgado, Alan Humason, Robert Kalescky, Marek Freindorf, Elfi Kraka

**Affiliations:** Computational and Theoretical Chemistry Group, Department of Chemistry, Southern Methodist University, 3215 Daniel Avenue, Dallas, TX 75275-0314, USA; alexisdelgado81096@gmail.com (A.A.A.D.); humason.alan@gmail.com (A.H.); rkalescky@smu.edu (R.K.); mfreindorf@smu.edu (M.F.)

**Keywords:** longest CC bonds, vibrational spectroscopy, local mode theory, local mode force constants, steric versus electronic effects

## Abstract

For decades one has strived to synthesize a compound with the longest covalent C−C bond applying predominantly steric hindrance and/or strain to achieve this goal. On the other hand electronic effects have been added to the repertoire, such as realized in the electron deficient ethane radical cation in its D${}_{3d}$ form. Recently, negative hyperconjugation effects occurring in diamino-o-carborane analogs such as di-N,N-dimethylamino-o-carborane have been held responsible for their long C−C bonds. In this work we systematically analyzed CC bonding in a diverse set of 53 molecules including clamped bonds, highly sterically strained complexes such as diamondoid dimers, electron deficient species, and di-N,N-dimethylamino-o-carborane to cover the whole spectrum of possibilities for elongating a covalent C−C bond to the limit. As a quantitative intrinsic bond strength measure, we utilized local vibrational CC stretching force constants $k^{a}$(CC) and related bond strength orders BSO *n*(CC), computed at the ωB97X-D/aug-cc-pVTZ level of theory. Our systematic study quantifies for the first time that whereas steric hindrance and/or strain definitely elongate a C−C bond, electronic effects can lead to even longer and weaker C−C bonds. Within our set of molecules the electron deficient ethane radical cation, in D${}_{3d}$ symmetry, acquires the longest C−C bond with a length of 1.935 Å followed by di-N,N-dimethylamino-o-carborane with a bond length of 1.930 Å. However, the C−C bond in di-N,N-dimethylamino-o-carborane is the weakest with a BSO *n* value of 0.209 compared to 0.286 for the ethane radical cation; another example that the longer bond is not always the weaker bond. Based on our findings we provide new guidelines for the general characterization of CC bonds based on local vibrational CC stretching force constants and for future design of compounds with long C−C bonds.

## 1. Introduction

Carbon-carbon single bonds are essential to organic chemistry and form the framework for many materials and life forms, e.g., serving as fundamental connectors in genes and proteins. The typical length of a C(sp^3^)−C(sp^3^) bond is around 1.54 Å [1]. However, it may exceed this standard value considerably, which has led to a competition of pushing the CC bonds to their limits by finding the best strategy for synthesizing the longest but still intact CC bond [2,3,4,5,6,7]. Whereas the question of the practical use of such ultra-long C−C bonds is still open, the study of these extreme cases including novel CC bonding situations such as e.g., the recently established CC tetrel bonding [8,9,10] strongly contributes to enriching our understanding of the CC bond and the concept of the chemical bond in general [11,12,13]. Besides long C−C bonds other elongated bonding situations are also of interest, notably long OO bonds and dative bonding. Interestingly, spectroscopic analysis of gas phase HOON has revealed that the O−O bond surpasses a length of 1.91 Å (1.9149 ±0.0005 Å) but is relatively stable than previously thought [14]. Also, computational analysis of H_2_O_6_, conducted at the CCSD(T)/cc-pVTZ level of theory, has shown the molecule to have an unusually long central O−O bond at a length of 1.91 Å (≈1.902 Å) [15]. Dative bonding within BN systems involves the substitution of two C atoms with boron and nitrogen atoms; in contrast to carbon analogs, longer and weaker bonds result for dative bonds. For instance, the B−N single bond of NB ethylamine, evaluated at the CC2/TZVPP level of theory, shows to be the strongest dative B−N bond and is 0.08 Å greater than the usual C(sp^3^)−C(sp^3^) bond length [16].

Increasing the *exchange (steric) repulsion* via bulky substituents has long been known to lengthen interatomic distances. In an alkane, one way to increase the bond length between two carbons is to replace hydrogens with alkyl groups [17,18,19,20,21,22,23,24]. The central C−C bond of 2,2,3,3-tetramethylbutane [25] was one of the first investigated in this regard, and many related studies followed [20,21,23,24,26]. Schreiner and co-workers [27] constructed dumbell-shaped molecules consisting of a central C-C bar holding on each end three-dimensional diamond-like alkanes (so-called diamondoids). The outer surfaces of these diamondoids are capped by hydrogens, the van der Waals attraction between the hydrogens on either side of the central bond holds these molecules together. Meanwhile, the repulsive forces of each diamondoid on either side of the central C−C bridge are sufficient enough to stretch the bond by more than 0.2 Å compared to the typical C−C bond length of an alkane. The enforcement of a cage-topology *clamped bonds*, involving the bridging atoms, is another way to lengthen a covalent bond [28,29]. Some extensively investigated examples regarding clamped bonds are bi(anthracene-9,10-dimethylene) [30] and acenaphthene-5,6-diyl bis(diphenylmethylium), both being sensitive to light, heat, and pressure [31]. Moret and co-workers discussed the formation of exceptionally weak C−C bonds by metal-templated pinacol coupling, where metal coordination is the key stabilizing factor [32]. A different strategy has been based on the fact that the loss of bonding electrons or bonding electron density leads to weaker and longer bonds as a consequence of electron deficient bonding. The D${}_{3d}$ symmetrical ethane radical cation is a classic example with calculated C−C bond lengths of 1.915 Å or more, depending on the level of theory used [33,34,35]. A series of diamino-o-carboranes have been synthesized with inner-cluster C−C bond lengths between 1.990 Å [4] and 1.931 Å [5] for which negative hyperconjugation between the nitrogen lone pairs and the $\sigma^{*}$(C−C) orbital has been held primarily responsible for causing CC bond elongation [3]. Recently, Mandal et al. demonstrated in a B3PW91-D3/cc-pVTZ study how fine tuning of negative hyperconjugation effects can result in even longer C−C bonds as in the case of amino oxide-o-carborane and di-N,N-dimethylamino-o-carborane [12].

In contrast to the vast number of experimental and theoretical studies, the relationship between CC bond elongation and the intrinsic strength of the CC bond is not well established, mainly caused by the lack of a reliable intrinsic bond strength measure which is required to systematically quantify the CC bond strength in these systems. Although bond dissociation energies (BDE)s and bond dissociation enthalpies (BDH)s play a fundamental role in determining chemical reactivity and selectivity [36,37,38] their use as bond strength measures is questionable. BDEs/BDHs are reaction parameters that include all changes taking place during the dissociation process. Accordingly, they include any (de)stabilization effects of the fragments to be formed, reflecting the energy needed for bond breaking, but also containing energy contributions due to geometry relaxation and electron density reorganization in the dissociation fragments. Therefore, the BDE/BDH is generally not a suitable measure for the intrinsic strength of a chemical bond and its use may lead to misjudgments, as documented in the literature [8,39,40,41,42,43,44,45]. Also the bond length is not always a qualified bond strength descriptor [46,47]. A handful of cases have been reported illustrating that a shorter bond is not always a stronger bond [48,49,50,51,52].

In this situation the local vibrational mode analysis (LMA), originally introduced by Konkoli and Cremer [53,54,55,56,57], offers an attractive alternative by providing local vibrational stretching force constants ($k^{a}$) as an ideal measure of the intrinsic strength of a bond and/or weak chemical interaction [58] including ultra long C−C bonds [11].

**Figure 1:**
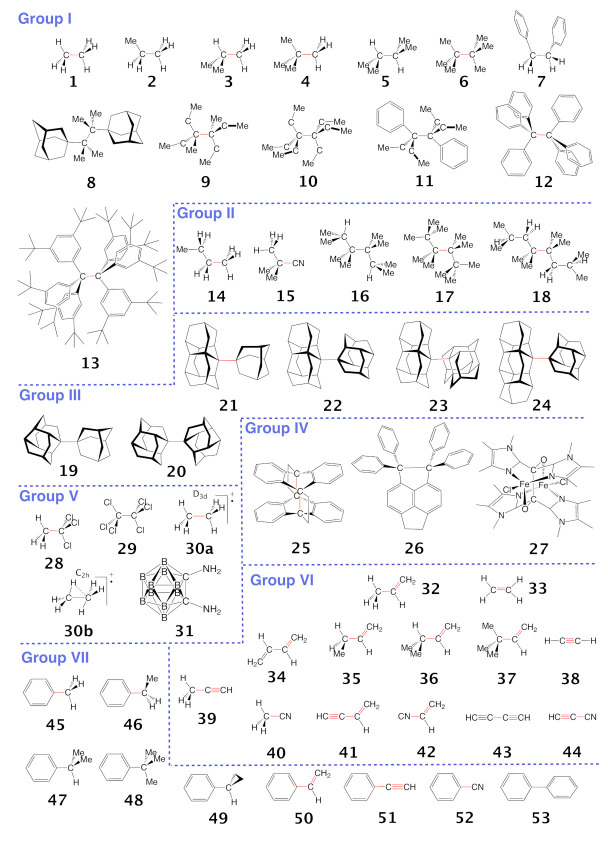
Molecules **1**–**53** investigated in this work, categorized in Groups I–VII. Targeted CC single, double, and triple bonds are shown in red.

We applied in this work LMA supported by natural bond orbital [59] and electron density [60,61] analyses to a diverse set of 53 molecules shown in Figure 1, possessing long C−C bonds representing distinct bonding scenarios to systematically assess the effect and interplay of steric, strain, and electronic factors leading to CC bond weakening. Molecules **1**–**53** are categorized into seven groups Group I–Group VII and targeted CC bonds are indicated by red coloration. The C−C bonds of Group I molecules (**1**–**13**) and Group II molecules (**14**–**18**) are exposed to increasing steric repulsion as the bulkiness of substituents increases. Group III molecules (**19**–**24**) represent different diamondoid configurations. Group IV molecules (**25**–**27**) represent both strained and clamped C−C bonds. Molecules **28**–**31** of Group V reflect electronic effects on the C−C bond. Group VI molecules (**32**–**44**) and Group VII molecules (**45**–**53**) contain conjugated C−C bonds and CC double and triple bonds used as reference.

## 2. Computational Methods

Equilibrium geometries and vibrational frequencies for all molecules were derived using the ωB97X-D functional [62,63] in combination with Dunning’s aug-cc-pVTZ basis set [64]. The ωB97X-D functional was chosen as it has proven to reliably describe weak (long-range) intermolecular interactions covering the diverse range of molecules considered [62,65,66,67]. The aug-cc-pVTZ basis set was applied as the augmented diffuse basis functions describe long range effects [68,69]. All DFT calculations were performed with the Gaussian 16 Rev. program package [70]. Geometry optimizations were conducted with an ultra fine grid integration and tight convergence criteria. BDHs were calculated for the target molecules using the Gaussian-4 (G4) composite method [71,72,73,74]. G4 is known for providing BDH values which are comparable to experimental results. Further, to verify the accuracy of the chosen methods, calculated C−C bond lengths and BDHs were compared with their experimental counterparts.

**Figure 2:**
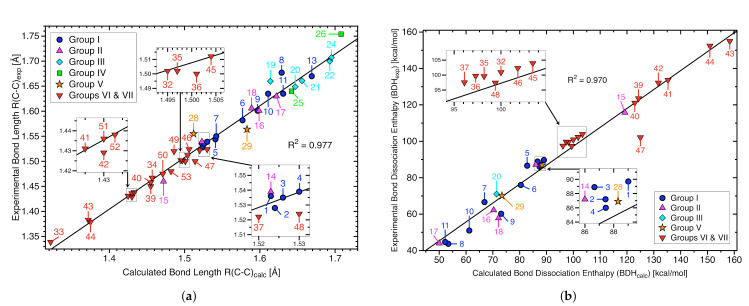
(**a**) Comparison between calculated and experimental C−C bond lengths for Group I–Group VII molecules (for available experimental bond lengths). The solid black line indicates a linear fit for the targeted C−C bonds; (**b**) corresponding correlation between BDH${}_{calc}$ and BDH${}_{exp}$ (for available experimental dissociation energies).

Considering the diversity of C−C bonds investigated in this work the agreement between calculated and experimental C−C bond lengths (Figure 2a, $R^{2}$ = 0.977) and calculated and experimental BDH values (Figure 2b, $R^{2}$ = 0.970) is satisfactory. It has to be noted that the experimental bond lengths were obtained by different techniques, at different temperatures, and in different environments leading to variations up to 0.035 Å. A comprehensive compilation of experimental bond lengths is provided in the Supporting Information, Table S1. This data includes electron diffraction, x-ray diffraction, microwave spectroscopy, and infrared spectroscopy data. A comprehensive summary of experimental BDH values are compiled in the Supporting Information, Table S2.

Second order perturbation stabilization energies ($\Delta E^{2}$), due to charge transfer events (i.e., orbital interactions), were retrieved via natural bond orbital (NBO) analysis through the application of the NBO6 program [75,76]. Electron densities ($\rho_{b}$) and energy densities ($H_{b}$) at CC bond critical points $r_{b}$ were determined with the AIMAll package [77]. The nature of the CC bonds was characterized following the Cremer-Kraka criterion, which implies that covalent bonding is characterized by a negative energy density, i.e., $H_{b}$ < 0 whereas electrostatic interactions are indicated by positive energy density values, i.e., $H_{b}$ > 0 [78,79,80]. Following geometry optimization and normal mode analysis, LMA was employed to quantify the intrinsic strength of the targeted CC bonds utilizing the LModeA software [58,81]. A comprehensive discussion of the underlying theory of LMA is provided in Reference [58], therefore in the following only a summary of the essential features are given.

Vibrational spectroscopy offers detailed information on the electronic structure of a molecule and its chemical bonds as encoded in the normal vibrational modes, ready to be deciphered. However, normal vibrational modes in a polyatomic molecule tend to couple, therefore they cannot be directly used to derive an intrinsic bond strength measure [82,83]. There are two coupling mechanisms between normal vibrational modes, *mass coupling* and *electronic coupling*. The electronic coupling can be eliminated via the Wilson GF-matrix formalism [82,83,84,85], i.e., by solving the secular equation of vibrational spectroscopy, which is a standard procedure in all quantum chemistry package calculating normal vibrational frequencies and corresponding normal modes. The vibrational secular equation expressed in terms of $N_{vib}=(3N-\Sigma$) internal coordinates q; (Σ: number of translations and rotations; 6 for nonlinear and 5 for linear molecules) is given by [82]

(1) $$F^{q}D=G^{-1}D\Lambda$$

$F^{q}$ is the force constant matrix expressed in terms of internal coordinates q, **G** is the Wilson **G** matrix [82], also called the “inverse kinetic energy” matrix. The eigenvector matrix **D** is comprised of vibrational eigenvectors **d**${}_{\mu }$ (μ = 1,...,$N_{vib}$) and the diagonal matrix Λ contains the vibrational eigenvalues $\lambda_{\mu }$ ($\lambda_{\mu }$ = 4$\pi^{2}c^{2}\omega_{\mu }^{2}$, *c* = constant for the speed of light and $\omega_{\mu }$ = harmonic vibrational frequencies of the normal mode vectors $d_{n}$ expressed in cm${}^{-1}$). Solving Equation (1) the diagonal normal force constant matrix $F^{Q}$ = K is obtained,

(2) $$F^{Q}=K=D^{†}F^{q}D$$

where Q is a vector that collects $N_{vib}$ normal coordinates [86]. It is important to note that the diagonalization of the force constant matrix $F^{q}$, i.e., transforming to normal coordinates Q [87,88,89] eliminates the off-diagonal coupling force constant matrix elements and in this way the electronic coupling [82]. However, it does not eliminate the kinematic (mass) coupling which often has been overlooked. Konkoli and Cremer solved this problem by introducing a mass-decoupled analogue of the Wilson equation [53,54,55,56] resulting in local vibrational modes ($a_{n}$) that are free from any mode-mode coupling

(3) $$a_{n}=\frac{K^{-1}d_{n}^{†}}{d_{n}K^{-1}d_{n}^{†}}$$

where $a_{n}$ represents the local mode vector that is affiliated with the *n*-th internal coordinate $q_{n}$ describing the local mode *n*. To each local mode **a**${}_{n}$ a local mode force constant $k_{n}^{a}$ can be assigned

(4) $$k_{n}^{a}=a_{n}^{†}Ka_{n}={\left(d_{n}K^{-1}d_{n}^{†}\right)}^{-1}$$

and a local vibrational frequency $\omega_{n}^{a}$

(5) $${\left(\omega_{n}^{a}\right)}^{2}=\frac{1}{4\beta^{2}c^{2}}k_{n}^{a}G_{n,n}^{a}$$

where $G_{n,n}^{a}$ corresponds to a diagonal element of the Wilson **G** matrix.

There is a 1:1 relationship between the normal vibrational modes and each complete, non-redundant set of local vibrational modes via an *adiabatic connection scheme* (ACS) [90], which can be considered as the most important milestone of the local mode theory; (i) it proves the physical relevance of local vibrational modes and (ii) it forms the basis for the decomposition of each normal mode into local mode contributions, providing a new comprehensive way to analyze vibrational spectra [58]. Any normal vibrational mode $l_{\mu }$ can be decomposed into local mode contributions [55,57] leading to a detailed analysis of the vibrational spectrum and a wealth of information about structure and bonding [91,92].

Local mode force constants, contrary to normal mode force constants are independent of the choice of the coordinates used to describe the molecule in question [90,93,94]. They are sensitive to differences in the electronic structure (e.g., caused by changing a substituent), and because they are, in contrast to frequencies, independent of the atomic masses, they capture pure electronic effects. In their landmark paper, Zou and Cremer [95] provided the important proof that the local stretching force constant $k_{n}^{a}$(AB) reflects the intrinsic strength of the bond/interaction between two atoms A and B being described by an internal coordinate $q_{n}$. Replacing the calculated vibrational frequencies in Equation (5) with measured fundamental frequencies leads to experimentally based local mode force constants [57] including anharmonicity effects not being captured by calculated harmonic force constants [91,96]. This important feature opens LMA to the experimental vibrational spectroscopists. For the comparison of larger sets of $k_{n}^{a}$ values, the use of a relative bond strength order BSO *n* is more convenient. Both are connected according to the generalized Badger rule derived by Cremer, Kraka, and co-workers [39,93] via the following power relationship:

(6) $$\mathrm{BSO}\;n=a{\left(k^{a}\right)}^{b}$$

(For simplification, in the reminder of the manuscript $k^{a}$ represents $k_{n}^{a}$ i.e., $k^{a}$ = $k_{n}^{a}$.) The constants *a* and *b* are calculated from $k^{a}$ values of two reference compounds with known BSO *n* values and the requirement that for a zero force constant the corresponding BSO *n* value is zero. For the CC bonds investigated in this work, ethane (**1**) and ethene (**33**) were used as references with assigned BSO *n* values of 1.0 and 2.0, respectively [48]. The ωb97xd/aug-cc-pVTZ model chemistry utilized in this work leads to *a* = 0.3135 and *b* = 0.8062, approximately.

## 3. Results and Discussion

**Figure 3:**
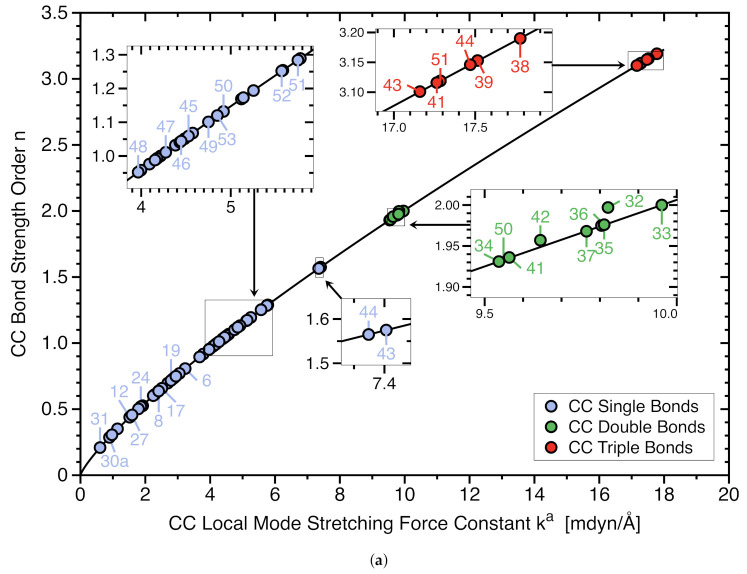

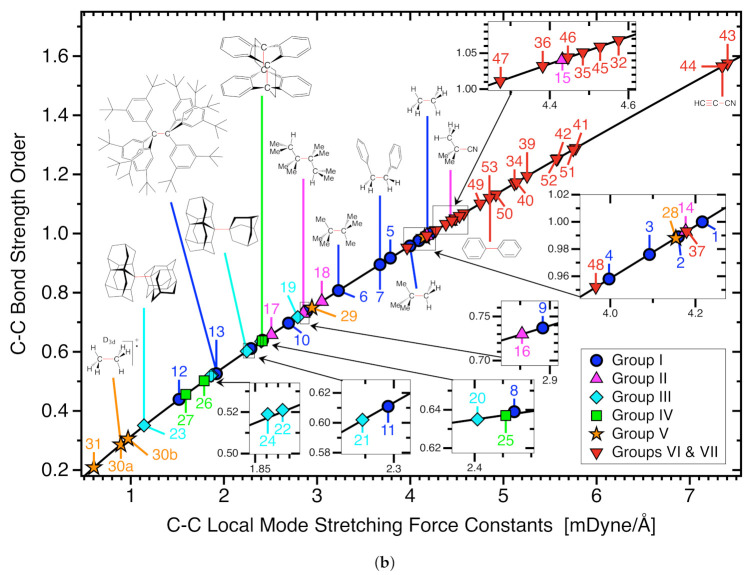
(**a**) BSO *n* values of all targeted CC single, double, and triple bonds for Group I–Group VII molecules, obtained with the power relationship BSO *n* = 0.3135 ($k^{a}$)${}^{0.8062}$ utilizing local mode stretching force constants $k^{a}$(CC). Calculated at the ωB97X-D/aug-cc-pVTZ level of theory. See text and Equation (6) for the derivation of the power relationship; (**b**) corresponding power relationship for all C−C bonds.

Table 1 summarizes the calculated CC bond distances (R${}_{calc}$), experimental CC bond distances (R${}_{exp}$), calculated bond dissociation enthalpies (BDH${}_{calc}$), experimentally determined bond dissociation enthalpies (BDH${}_{exp}$), local stretching force constants ($k^{a}$), local mode vibrational frequencies ($\omega^{a}$), bond strength orders (BSO *n*), electron densities ($\rho_{b}$), and energy densities (H${}_{b}$) for the targeted CC bonds of molecules **1**–**53**. Figure 3a shows the BSO *n* values and local stretching force constants $k^{a}$ for all targeted CC bonds of molecules **1**–**53** and Figure 3b exhibits the corresponding values for the targeted C−C bonds.

As depicted in Figure 3a the CC single bonds of our set of 53 molecules show the largest variation in bond strength (BSO *n*(C−C) values from to 0.21 to 1.58) reflecting the diversity of our test set whereas as expected, the variation of the bond strength of the double bonds (BSO *n*(C=C) values from 1.93 to 2.00), and that for the triple bonds is relatively small (BSO *n*(C≡C) values from 3.10 to 3.15), in line with the fact that is more difficult to modulate a CC double and triple bond than a CC single bond. The gap between single and double bonds (0.35 BSO *n* units) is smaller than that between double and triple bonds (1.10 BSO *n* units), confirming that the power relationship is in compliance with general organic chemistry rules.

The focus in Figure 3b is on CC single bonds. As expected, BSO *n* values larger than one (ethane, **1**) are found for Group VI and VII members with conjugated C−C bonds. 1,3-butadiyne **43** and cyanoethyne **44** have the strongest C−C bonds with BSO *n* values of 1.575 and 1.565, respectively. The middle range (BSO *n* values between 0.5 and 1) representatives of Group I - Group V are found, revealing that different scenarios can lead to CC bond elongation. On the low end of the bond strength spectrum (BSO *n* values > 0.5) the diiron(II) bis(1-methyl-4,5-dimethylimidazolyl)pinacolate bis-chlorido complex **27** (BSO *n* = 0.456, weakest C−C bond within Group IV), hexaphenylethane **12** (BSO *n* = 0.439, weakest C−C bond within Group I), 2-(2-triamantyl)triamantane **23** (BSO *n* = 0.351, weakest C−C bond within Group III) are located. The overall weakest C−C bonds are found for Group V members representing electron deficient bonding; ethane radical cation D_3d_ **30a** (BSO *n* = 0.286) and C_2h_ **30b** (BSO *n* = 0.306) and di-N,N-dimethylamino-o-carborane **31** (BSO *n* = 0.209). It has to be noted that whereas **31** has the weakest C−C bond, **30a** has the longest C−C bond of 1.935 Å compared with the R(C−C) of 1.930 Å for **31**.

**Figure 4:**
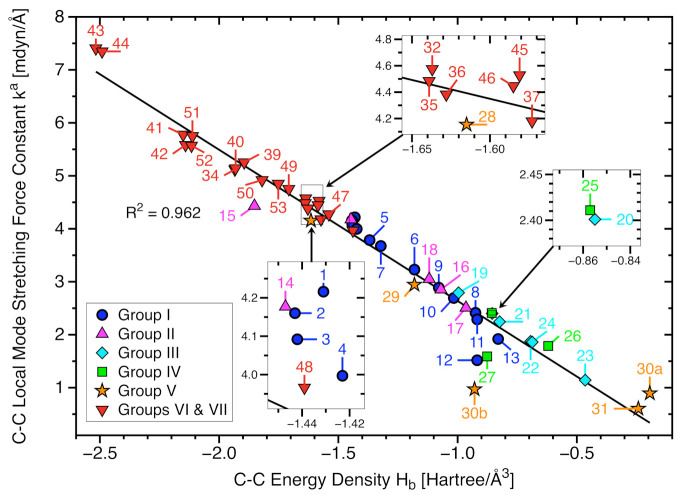
Local mode stretching force constants $k^{a}$(C−C) versus energy density $H_{b}$ for all C−C bonds (Groups I through VII). ωB97X-D/aug-cc-pVTZ level of theory.

Figure 4 confirms that all targeted C−C bonds of our set of 53 molecules, including the weakest such as **30a** and **31**, are covalent in nature according to the Cremer-Kraka criterion of covalent bonding, i.e., all $H_{b}$ values are smaller than zero (see also Table 1). The correlation between the local stretching force constants $k^{a}$ of targeted C−C bonds and the corresponding energy density $H_{b}$ is moderate ($R^{2}$ = 0.962), reflecting that the local force constant picks up the electronic environment of a bond whereas $H_{b}$ reflects the electronic structure just at a single point, i.e., the bond critical points **r**${}_{b}$. [58]. Nevertheless, a general trend can be visualized; the C−C bond strength decreases alongside a decrease in covalent character.

### 3.1. Bond Dissociation Enthalpies and Bond Lengths as Bond Strength Measure

**Figure 5:**
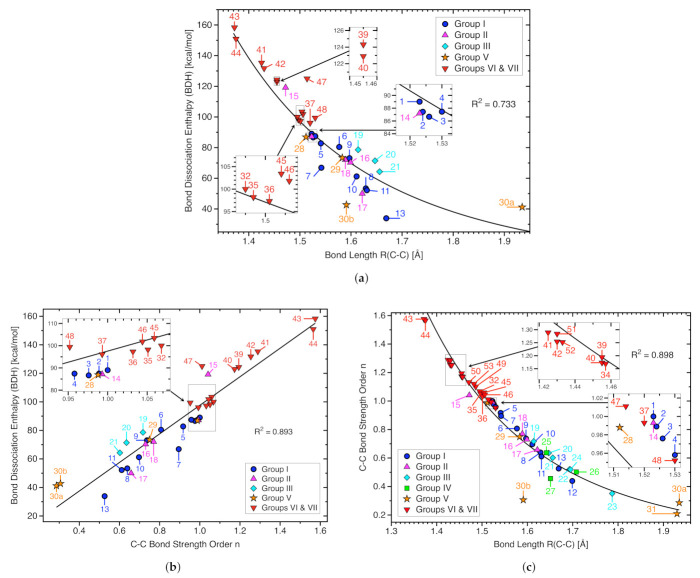
(**a**) Correlation between calculated bond dissociation enthalpies $BDH_{calc}$ and calculated C−C bond lengths. BDH values calculated with the composite G4 method and R(C−C) values at the ωB97X-D/aug-cc-pVTZ level of theory; (**b**) correlation between $BDH_{calc}$ and BSO *n*(C−C). $BDH_{calc}$ values were obtained using G4 and BSO *n*(C−C) values calculated at the ωB97X-D/aug-cc-pVTZ level of theory; (**c**) Relationship between BSO *n*(C−C) and R(C−C) calculated at the ωB97X-D/aug-cc-pVTZ level of theory.

Zavitsas [103] reported a linear relationship between CC bond lengths and bond dissociation energies (BDE)s for strained and unstrained compounds, which predicts a maximum C−C bond length of 1.75 Å. However, this simple relationship fails for C−C bonds in more complex situations, such as for compounds with extraordinary ring strain or steric congestion [19,51,126]. In Figure 5 the corresponding calculated BDH and R(C−C) values for our set of 53 molecules are compared. In contrast to Zavitsas, we find a qualitative exponential relationship ($R^{2}$ = 0.733) with several outliers such as sterically crowed molecules **7** and **13** in line with previous observations [19,51,126] and the ethane radical cations **30a** and **30b**. In particular **30b** with a bridging hydrogen bond situation falls off the curve.

In Figure 5b $BDH_{calc}$ values are correlated with the C−C bond strength orders BSO *n*(C−C). Although there is some trend that the BDH value increases with increasing C−C bond strength, the overall correlation is moderate (R${}^{2}$ = 0.893), showing that the BDH is not a good measure for the intrinsic strength of the CC bond because it includes the overall effects of bond breakage, as discussed in the introduction. This concerns in particular crowded molecules with larger possibilities for geometry and electron density reorganization of the fragments such as **7**, **13**, and **15**. Figure 5c correlates BSO *n*(C−C) values with calculated C−C bond lengths. Again there is some trend that shorter C−C bonds are stronger bonds (R${}^{2}$ = 0.898). However there are also some outliers, such as the organometallic compound **27** and the ethane radical cation **30b** representing the special case of hydrogen bridging. This clearly shows that a comprehensive discussion of the intrinsic CC bond strength is better based on local vibrational modes and corresponding bond strength orders, which is pursued in the following sections focusing on trends within the individual groups.

### 3.2. C−C Bonds of Group I

The central C−C bonds of Group I molecules (**1**–**13**) are subjected to increasing levels of steric congestion as substituent bulkiness is incremented from **1** to **13**. The C−C bond lengths for molecules **1**–**11** and **13** range between 1.523 Å to 1.669 Å, where the shortest C−C bond length is exhibited by ethane (**1**). The C−C bond lengths mentioned above are consistent with experimentally derived values (See Table 1 and Figure 2a). As the methylation of ethane proceeds from **1** to **6** the elongation of the C−C bond progresses from 1.523 (**1**) to 1.577 mdyn/Å (**5**) alongside bond weakening depicted by decreases in BSO *n* values from 1.000 (**1**) to 0.807 mdyn/Å (**6**) (See Figure 3). The overall stabilization energy values ($\Delta E^{2}$), due to the hyperconjugation (i.e., charge transfer) predominately occurring from σ(C−H) →$\sigma^{*}$(C−C), reveal increasing hyperconjugation as the methylation of ethane proceeds from **1** to **6** ($\Delta E^{2}$ = 0.00 (**1**), 3.73 (**2**), 7.74 (**3**), 13.29 (**4**), 14.84 (**5**), 28.14 kcal/mol (**6**)). Further, the orbital occupancy (e) of the central σ(C−C) orbital of molecules **1**–**6** decreases as the methylation proceeds (1.997 to 1.960 e) while the occupancy of the $\sigma^{*}$(C−C) orbital steadily increases (0.00 to 0.05 e). Thus, the target C−C bonds of **1**–**6** increase and decrease in length and strength due to increasing hyperconjugation between σ(C−H) and $\sigma^{*}$(C−C), the increase in hyperconjugation is responsible for the parallel relationship between the decrease of σ(C−C) electron occupancy and increase of $\sigma^{*}$(C−C) electron occupancy.

The C−C bond lengths for molecules **5** and **7** are virtually the same with BSO *n* values of 0.917 ($k^{a}$ = 3.786 mdyn/Å) and 0.895 ($k^{a}$ = 3.675 mdyn/Å). It is noted that the *gauche* configuration for 1,2-diphenylethane (**7**) results in the most stable rotational isomer (by 1.10 kcal/mol); this result indicates the presence of π-stacking which may contribute towards the shortening of the central C−C bond. Moreover, the central C−C bond of **7** is longer than that of **5** as the steric repulsion between the two phenyl groups of **7** outweighs that which occurs between the four methyl groups of system **5**. The C−C bond of **6**, in comparison to that of **5** and **7**, is forced to a greater distance due to the steric crowding of the six methyl groups (See Table 1). Further, the electron density value ($\rho_{b}$) at the C−C bond critical point of **6** is less than that of both **5** and **7** while the $\rho_{b}$ of **5** is greater than that of **6** and **7** ($\rho_{b}$ = 1.645 (**5**), 1.544 (**6**), 1.601 e/Å${}^{3}$ (**7**)). The C−C bond of molecule **6** is longer and weaker than that of **5** and **7** as a result of weaker covalent bond character indicated by a less negative energy density value at the C−C bond critical point ($H_{b}$ = −1.369 (**5**),−1.181 (**6**), −1.322 h/Å${}^{3}$ (**7**)), the decrease in the covalent bond character is reflected from a smaller σ(C−C) occupancy (1.963 (**5**), 1.960 (**6**), and 1.974 e (**7**)) together with a larger $\sigma^{*}$(C−C) occupancy (0.028 (**5**), 0.050 (**6**), and 0.021 e (**7**)).

Targeted C−C bonds within molecules **8**–**13** range in length between values of 1.597 and 1.699 Å as BSO *n* measures fluctuate amidst values of 0.439 and 0.737, where the C−C bond length elongates as the bond strength decreases. The repulsive forces between the adamantane groups of **8** enable the C−C bond length to become longer in contrast to molecules **9** and **10**, where methyl and/or only ethyl groups are present; the repulsive forces between substituents of **9** and **10** do not exceed that occurring between the adamantane groups of **8** as reflected by the shorter C−C bond length values for **9** and **10** (R = 1.629 (**8**), 1.597 (**9**), 1.611 Å (**10**)). The steric repulsion between the phenyl and ethyl substiuents of **11** is greater than that occurring between adamantane substituents of **8** as indicated by from the longer C−C bond length for **11** (R = 1.631 Å). The electron density values of **8**–**13** decrease in parallel to C−C bond lengthening and weakening (see Table 1) as well as the magnitude of corresponding energy density values, these results shows that longer and weaker C−C bonds concur as C−C covalent bond character diminishes (see Table 1 and Figure 2b).

Molecules **12** and **13** involve complete phenylation of ethane, in the case of **13** methyl groups replace two hydrogen atoms on every phenyl group, increasing steric crowding even further. In contrast to **13**, which has been synthesized and characterized [106,127], **12** has not been isolated so far being in line with the small calculated BDH value of 16.6 kcal/mol compared to that of **13** (BDH = 33.9 kcal/mol). It seems counter-intuitive that for the less crowded hexaphenylethane **12** a longer C−C bond is found than for the hexakis-(3,5-di-tert-butylphenyl)ethane **13**, R = 1.699 versus 1.669 Å and that **13** is more stable. Schreiner et al. [127] attribute the stabilization of the more crowed compound to London dispersion through the increasing polarizability of alkyl substituents and the fact that conformationally more flexible hydrocarbon substituents introduce large unfavorable entropy contributions which help to stabilize these extraordinary bonding situations. This clearly shows that steric crowding, although being a key factor for CC bond elongation, strongly depends on the bonding environment. As a consequence, the introduction of even more sterically demanding substituents, such as adamantyl groups, could even lead to a further reduction of the central C−C bond length instead of the desired elongation.

### 3.3. *C−C Bonds of Group II*

Molecules **14**–**18** obtain C−C bond lengths ranging between values of 1.472–1.787 Å where BSO *n* values vary between 0.604–0.993. The di-methylation of ethane in a trans configuration (**14**) results in a slightly shorter bond than that of the mono-methylated equivalent (**2**) (See Table 1). Molecule **14** obtains a slightly shorter and stronger than **2** due to a slightly greater bond covalent character ($H_{b}$ = −1.443 (**2**), −1.447 (**14**) Hartree/Å${}^{3}$) attributed to the larger amount of allocated electron density at the C−C bond critical point (See Table 1). Comparing the C−C bond lengths within systems **4** (2-methylpropane) to **15** (2-cyano-2-methylpropane) reveals that the the replacement of three hydrogen atoms with a nitrogen atom causes the C−C bond to shorten from 1.530 to 1.472 Å, both bond length values being in good agreement with experimental results (See Table 1). The nitrogen atom of **15** results in C−C charge values of −1.160 and +0.295 e, where the positive charge value is that of the carbon atom directly linked to the nitrogen atom (See Figure S1, Supporting Information). Because NBO C−C charges for **4** reflect repulsion between C−C atoms, unlike **15** where the charges denote attraction between CC atoms, the results reveal that the inductive through bond effect of nitrogen results in a the polarization of the C−C bond; the polarization of this bond causes the C−C bond to shorten further due to attractive forces between the carbon atoms. As a result of the attractive forces between carbon atoms of C−C within **15**, with respect to that of **4**, a larger covalent bond character results ($H_{b}$ = −1.423 (**4**), −1.852 Hartree/Å${}^{3}$ (**15**)). Moreover, the C−C bond of **15** obtains a greater value of electron density at the C−C bond critical point in contrast to **4** (See Table 1). The enhancement of the covalent bond character for C−C bond of **15** causes the CC bond of **15** to be stronger than that of **4** (BSO *n*: 0.958 (**4**), 1.787 (**15**)).

The application of 4 methyl and 2 isopropyl groups (**16**) and 4 methyl and 2 *tert*-butyl groups (**17**) increases steric strain in contrast to the other systems of Group II, the increase in strain is reflected from longer C−C bond lengths of 1.599 (**16**) and 1.622 Å(**17**) which are the longest C−C bonds observed for Group II. Further, extending the carbon chain from six to eight carbons for a system with 4 methyl and 2 isopropyl groups, as represented by molecules **17** (2,2,3,3,4,4,5,5-octamethylhexane) and **18** (2,4,4,5,5,7-hexamethyloctane), results in a decrease of strain between functional groups (R(C−C) = 1.622 (**17**), 1.589 Å (**18**)). In general, C−C electron density values of **14**–**18** decrease alongside C−C bond elongation and weakening (see Table 1); as the electron density values decrease the energy density values $H_{b}$ at the C−C bond critical point become less negative. Thus, as for Group I, the C−C bonds for Group II molecules become weaker and longer as bond covalency decreases (See Table 1 and Figure 2b).

### 3.4. C−C Bonds of Group III

Fokin, Schreiner, and others [27,128,129] synthesized, characterized, and computationally investigated a series of homo and heterodimeric diamondoid compounds connected by exceptionally long C−C bonds. These adamantyl dimers are exposed to the inherent conflict of repulsion between the two extremely bulky groups only held together a single C−C bond. Despite the exceptionally long central bond C−C, these compounds are found to have exceptional stability, persisting at temperatures greater than 200 ${}^{\circ }$C which is primarily attributed to attractive London dispersion forces between these units caused by weak interactions between the hydrogen atoms on the cages on either side of the molecule [52,128,129]. Furthermore, the radical fragments resulting from homolytic cleavage are only capable of little geometry relaxation and rehybridization qualifying them as perfect test cases for exploring bond length bond dissociation energy relationships and challenging Zavitsas’ empirical relationship [103]. We included 1-(1-adamantyl)diamantane, 1-(1-diamantyl)diamantane, 2-(1-adamantyl)triamantane, 2-(1-diamantyl)triamantane, 2-(2-triamantyl)triamantane, and 2-(1-diamantyl)[121]tetramantane molecules (**19**–**24**) in this work, calculated with a larger basis set than previously published. Our model chemistry reproduces computational results well with one exception, compound **23**, a theoretical compound with a record C−C bond length of 1.830 Å, which has evaded synthesis so far. However, the previously reported molecule **23** turns out to be a transition state of first order at our level of theory. There exists a more stable rotational isomer with a C−C length of 1.787 Å , which is −17.8 kcal/mol lower in energy, see Table 1. Although the C−C bond lengths of this isomer is 0.043 Å longer than that of the transition state, it still remains the longest C−C bond found for this group.

The calculated C−C bond lengths for complexes **19**–**22** and **24**, ranging from 1.614 to 1.695 Å , are in good agreement with the experimental values, deviations range between 0.002–0.014 Å (see Figure 2b). As the sizes of interacting groups take up more space steric strain increases and is counterbalanced by increasing the C−C bond lengths as to maintain molecular stability. As C−C bonds of Group III molecules (**19**–**24**) become longer they become weaker as reflected by the local mode force constants which reduce from 2.792 mdyn/Å (molecule **19**) to 1.142 mdyn/Å (molecule **23**) and the corresponding BSO *n* values of 0.717 for **19** and 0.351 for **23**. The weakening of the C−C bonds is in line with progressive bond destabilization observed via the $H_{b}$ values ranging from −0.996 Hartree/Å${}^{3}$ for **19** to −0.465 Hartree/Å${}^{3}$ for **23**. It has to be noted that the strained adamantyl dimer **23** outperforms the strained hexaphenylethane **12** of Group I in terms of a longer C−C bond (R = 1.787 versus 1.699 Å ) and weaker C−C bond (BSO *n* = 0.351 versus 0.439). Overall these two groups show similar CC bond elongation effects as depicted in Figure 3b, outperforming Group II members.

### 3.5. C−C Bonds of Group IV

The members of this group are bianthracene-9,1-dimethylene, molecule **25**, 1,1,2,2-tetraarylpyracene, molecule **26** in which one of the aliphatic five-membered rings (5MR)s annealed to a naphthalene chromophore is substituted by four bukly phenyl groups, and the diiron(II) bis(1-methyl-4,5-dimethylimidazolyl)pinacolate bis-chlorido complex, molecule **27**. They represent a situation where the central C−C bond can no longer freely expand to counterbalance steric crowding because it is “clamped” in the molecular framework. Despite this obvious restriction, molecules **25**–**27** compete well with their Group I and Group III counterparts, as shown in Figure 3b. It also has to be noted that homolytic C−C bond breakage in these systems is hampered by the fact that the two fragments cannot easily separate, potentially leading to recombination. Therefore, it has been suggested that the bonded and non-bonded states in these systems are seamlessly connected in terms of the interatomic C−C distances [7].

Molecule **25** has a calculated C−C bond length of 1.642 Å. The result agrees well with the experimental value of 1.640 Å, obtained by Dougherty et al. [108], as well as a previously calculated value of 1.645 Å [30]. For **26**, the calculated C−C length (1.708 Å) is much shorter than the experimental value of 1.754 Å, which was obtained via low temperature X-ray crystallography [7,31]. As discussed by the authors, this discrepancy could result from a triplet diradical with a considerably larger CC bond distance (3 Å) being in thermal equilibrium with the closed shell structure in the crystal at higher temperatures.

In line with the bond lengths the C−C bond of **25** is somewhat stronger than that of **26**, as reflected by the BSO *n* values of 0.637 and 0.502, respectively. The NBO charges of the C atoms composing the C−C bond in **26** are slightly larger than those of C atoms composing the C−C bonds in **25** (−0.185 e versus −0.044 e respectively, see Figure S1, Supporting Information) leading to larger repulsive forces between C−C atoms in **26** adding to the larger steric congestion caused by the four phenyl substituents. Furthermore, the molecular framework of **26** allows the two carbon atoms to separate more than it is possible in **25** by widening the CCC bond angles at the C−C base of the substituted 5MR to 109.3 ${}^{\circ }$ compared to 106.9 ${}^{\circ }$ in the unsubstituted 5MR with a much smaller effect on CCC angle opposite to base, 116.3 ${}^{\circ }$ compared to 116.0 ${}^{\circ }$ for the unsubstituted 5MR. In line with observed bond length trends are the energy density values for **25** and **26** suggesting that the C−C bond of **26** is less covalent bond nature than the corresponding C−C bond of **25**; ($H_{b}$ = −0.620 Hartree/Å${}^{3}$ for **26** and −1.857 Hartree/Å${}^{3}$ for **25**). These two examples clearly show the impact of the molecular framework on CC bond elongation.

Molecule **27** complements the group with organometallic compounds. As recently reported, Fe and Zn templated pinacol-type coupling leads to dinuclear metal complexes with ultra long central C−C bonds (R > 1.7 Å) being clamped by the organometallic framework [32,130]. For the diiron(II) bis(1-methyl-4,5-diphenylimidazolyl)pinacolate bis-chlorido complex a central C−C bond length of 1.730 Å was confirmed by both Xray structure and DFT calculations [32], which is much longer than a usual pinacolate type C−C [131]. It has been suggested that metal coordination contributes to stabilizing the weak C−C bond in the obtained cage structure. In order to make the calculations more feasible we simplified the complex by replacing the eight phenyl groups with methyl groups leading to the corresponding bis(1,4,5-trimethylimidazolyl)pinacolate complex **27**, see Figure 1. This resulted in a stable molecule, with a C−C bond length of 1.651 Å, in close agreement with the C−C bond length of the protonated pinacolate compound discussed by Folkersma et al. [32]. The relatively large positive charge of 0.210 e (see Figure S1, Supporting Information) assists in pushing the two carbon atoms apart. We find a BSO *n* value of 0.456, identifying the targeted C−C bond in **27** as the weakest in Group IV showing the strong potential of organometallic frameworks hosting ultra long C−C bonds.

### 3.6. C−C Bonds of Group V

The overall theme of Group V systems is CC bond elongation via electron deficient bonding. Molecules **28**–**29** were chosen to evaluate if electron withdrawing halogen substituents can lead to CC bond elongation. **30** exploits a more drastic option, i.e., electron removal leading to a radical cation, and molecule **31** combines the clamped CC situation with negative hyperconjugative effects.

To evaluate electron withdrawing effects on the C−C bond length chlorinated ethane molecules **28** and **29** were investigated. For **28** we find a C−C bond that is 0.011 Å shorter than the corresponding C−C bond in **1**. This value is in perfect agreement with the experimental value supporting the empirical rule of Sugie et al. [109], which suggests that addition of Cl atoms shortens generally a C−C bond. As reflected by the NBO charges of **28** (−0.638 e for the CH_3_ carbon versus −0.099 e for the CCl_3_ carbon as compared to −0.585 e for the carbon atoms of **1**, see Figure S1, Supporting Information) chlorination of one methyl group leads to a polar C−C bond. However, although being shorter, the resulting C−C bond becomes weaker as reflected by a $k^{a}$ value of 4.154 mdyn/Å compared to 4.216 mdyn/Å for **1**, whereas the $H_{b}$ value of −1.615 Hartree/Å${}^{3}$ compared to −1.431 Hartree/Å${}^{3}$ for **1** is in line with bond shortening. It is important to note that the local mode force constant as a second order property is a sensitive bond strength measure picking up subtile second order effects which are not shown in the energy density evaluated just at the bond critical point which describes the covalent character, whereas $k^{a}$ includes counterbalancing steric repulsion effects or C−C bond weakening via delocalization of charge from the Cl lone pair into the $\sigma^{*}$(C−C, i.e., the so-called negative hyperconjugation effect [132,133], or intramolecular halogen-halogen dispersion interaction [134]. Further details on the systematic chlorination of **1**, including calculations with a DFT functional designed for the description of dispersion effects, is included in the Supporting Information. Full chlorination of ethane, as realized in hexachloroethane **29**, results in C−C bond lengthening of 0.06 Å compared to **1**, revoking Sugie’s rule [109]. The steric congestion of the six Cl atoms overrides the electron withdrawing effects showing that electronic effects may not always cooperate with steric effects. Therefore, a case by case investigation is necessary and the local mode force constant can serve as a helpful guide.

The next Group V representative is the ethane radical cation in which one electron is removed from **1** either out of the outer valence shell of the C−C or the C−H orbitals resulting in a diborane-like isomer of C${}_{2h}$ symmetry, molecule **30b** which is slightly lower in energy of 0.3 kcal/mol than a second isomer **30a** with D${}_{3d}$ symmetry [33,135,136]. In line with experimental data, we find that the C−C bond length of 1.591 Å is still close to that in **1**. This is caused by H-bridge bonding, as revealed by the small C-C-H angles of 83.1${}^{\circ }$ of the in-plane H-atoms. This leads to a unique CC bonding situation which is reflected by the fact that in most graphs shown in this work **30b** appears as outlier as for example in Figure 4. In contrast, **30a** yields an ultra-long bond of 1.935 Å, the longest C−C bond found for all molecules investigated in this work as also reflected by the smallest of $H_{b}$ value of −0.194 Hartree/Å${}^{3}$. Intrigued by the strong bond lengthening effect of electron removal we also tested if this can be further increased by invoking steric strain. Therefore, we investigated potential radical cationic forms of **21** and **26**. Upon removal of an electron, the diamondoid system **21** dissociates into two fragments. In the clamped radical cation **26**, the single electron is delocalized over the entire molecule resulting in an increase of the C−C bond length from 1.708 Å to 1.709 Å , which cannot be considered meaningful. This example clearly shows that CC bond lengthening effects result from a complex interplay of electronic and steric effects and are not necessarily additive.

Di-N,N-dimethylamino-o-carborane, molecule **31** represents an interesting case for a clamped C−C bond which is, in addition, exposed to negative hyperconjucation. Li, Müller at al. synthesized a series of 1,2-diamino-o-carboranes with exceptionally long C−C bonds ranging between 1.627 and 1.931 Å depending on the amino substituents [5]. The longest C−C bond of 1.931 Å was found for the diphenyl compound. As a major factor for the CC bond elongation negative hyperconjucation between the nitrogen lone pair and the C−C $\sigma^{*}$ orbitals was identified [5]. Recently, Mandal et al. calculated an even longer C−C bond of 2.011 Å using a B3PW91-D3/cc-pVTZ model chemistry [12] which seems to overshoot the C−C bond length [137]. We find a C−C bond length for **31** of 1.930 Å, slightly shorter by 0.005 Å than the C−C bond in the ethane cation **30a** and in good agreement with the experimental data of Li, Müller at al. [5]. It is interesting to note that obviously two different effects, namely electron removal in **30a** and a clamped C−C bond exposed to negative hyperconguation in **31** leads to a comparable CC bond elongations. However, this is not true for the bond strength as reflected by the local mode force constants ($k^{a}$ = 0.894 mdyn/Å and BSO *n* = 0.286 for **31** compared with 0.604 mdyn/Å and BSO *n* = 0.209 for **30a**) qualifying the C−C bond in **31** as the weakest of all molecules investigated in this work.

### 3.7. CC Reference Bonds, Group VI and VII

Group VI and VII members comprise a series of molecules with hyperconjugated C−C bonds making them shorter and stronger as well as double and triple reference bonds. These molecules are included in order to validate the applicability of $k^{a}$(C−C) as a bond strength measure stretching from long to short C−C bonds. The C−C bonds in Group VI are in conjugation with a vinyl group and an alkyne (molecules **32**, **34**–**37**) or a cyano group (molecules **39**–**44**). Group VII C−C bonds, molecules **45**–**53**, are in conjugation with a phenyl group (see Figure 1).

The C−C bond lengths of Group VI and Group VII molecules (**32**–**52**) range from 1.372 to 1.530 Å, where the shortest and longest C−C bond lengths are reflected by molecules **43** and **48**. The C−C bond lengths for molecules of Groups VI and Group VII fall in line with experimentally determined measures (See Table 1 and Figure 2a). The BSO *n*(C−C) values Group VI and Group VII molecules (**32**–**52**) range from 0.952 to 1.575, where the weakest and strongest C−C bonds belong to molecules **43** and **48**. The shorter C−C bond of **43** obtains the largest value of energy density allocated at the C−C bond critical point region with regard to the targeted C−C bonds of molecules composing Groups VI and VII (See Table 1). Moreover, the C−C bond of molecule **43** is shorter and stronger than all other targeted C−C bonds for molecules of Groups VI and VII as **43** possesses the largest covalent C−C bond character (See Table 1). System **48** involves a trimethylated carbon attached to a carbon atom of a phenyl group, due to the presence of three methyl groups, which take up the most space out of all other systems of Groups VI and VII, the steric strain between the groups increases greatly in contrast to the other systems of Groups VI and VII. The increase in steric strain within **48** is mirrored by the elongation of the C−C bond, which enables **48** to possess the longest C−C bond for Groups VI and VII. Moreover, the C−C electron density and energy density at the C−C bond critical point is the smallest and the least negative (See Table 1). Thus, the longest and weakest C−C bond of Groups VI and VII, belonging to **48**, is due to **48** having the smallest C−C bond covalency as reflected by the least negative value of the energy density H${}_{b}$ for these groups (See Table 1).

**Figure 6:**
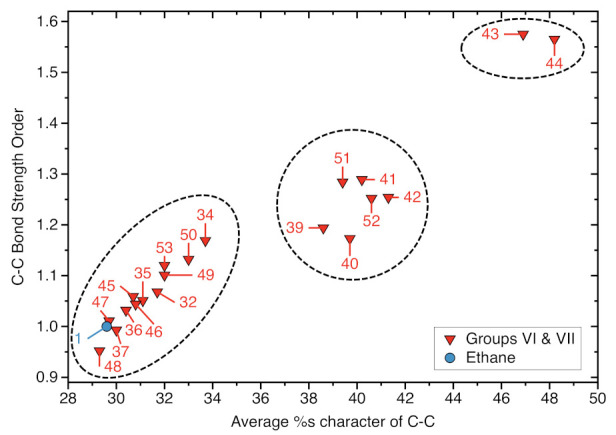
Average % s-character the targeted C−C bonds of ethane (**1**), Group VI, and Group VII molecules determined from the NBO analysis versus the corresponding BSO *n*(C−C) values. ωB97X-D/aug-cc-pVTZ level of theory.

A well-accepted concept in organic chemistry relates the length and the strength of a CC bond to its s-character, resulting from the type of hybridization. The more s character, the shorter and stronger the bond [138]. The average % s-character of a C−C single bond with two sp${}^{3}$ hybridized C atoms is 29.6, that of a C=C bond with two sp${}^{2}$ hybridized C atoms is 40.7, and that of a C≡C triple bond 52.3 in line with decreasing bond length and increasing bond strength in this order. Although this is a simplified picture, we see an interesting trend between the average %s character of the two C atoms forming the targeted bond and the local mode force constant as depicted in Figure 6 and Table 2 As the average % s character of the targeted C−C bond increases the C−C bond strength increases, adding to the model quantity % s character a physical relevance.

Furthermore, we observe a pattern with three clusters; the first cluster ranging from 28 to 35 average %s character involves systems where one of the targeted C atoms is bonded to a hydrogen and or methyl substituents and the other C atom bonded to a vinyl or only phenyl substituent (See Figure 6). The second cluster, where the average %s character of the C−C bond ranges from 37 to 43 %, includes systems in which one of the targeted C atoms is connected to an alkyne or cyano group (See Figure 6). The third cluster features systems **43** and **44** with the largest average %s characters of 46.9 % for **43** and 48.2 % **44**, respectively. In **43** both bond targeted C atoms are conjugation with an alkyne group and in **44** one of the targeted C atoms is conjugated with an alkyne and the other with a cyano group. Work is in progress to explore this further.

## 4. Conclusions and Outlook

In this work we investigated a diverse set of 53 molecules with ultra long C−C bonds including C−C bonds in a highly sterically strained situation, i.e., clamped C−C bonds, crowded bonding environment as found for diamondoid dimers, and electron deficient C−C bonds caused by electron removal, electron withdrawing substituents, or negative hyperconjugation as discussed for di-N,N-dimethylamino-o-carborane (organized in Groups I-V) to elucidate the wide spectrum of possibilities for elongating a covalent C−C bond. For comparison, we included also some C−C bonds within conjugated and hyperconjucated systems, which generally leads to shorter bonds, as well as a number of double and triple bonds (Groups VI and VII).

Vibrational spectroscopy provides a comprehensive overview of a molecule’s electronic structure and its local environment (e.g., the strength of intermolecular interactions) as concealed within the normal vibrational modes. We utilized local mode force constants, derived from local mode analysis, as a quantitative measure to assess the strength of these long C−C bonds. The local mode analysis was complemented with NBO analysis and electron density analysis where the covalent character of each ultra long C−C bond was confirmed via the energy density $H_{b}$ taken at the bond critical point **r${}_{b}$**. This has led to the following findings:

1. Although steric crowding/strain increases the C−C bond length electronic effects can lead to even longer and weaker CC bonds. The overall weakest C−C bonds are found within Group V where electron deficient bonding is represented by the ethane radical cation in D_3d_ (**30a**) (BSO *n* = 0.286) and C_2h_ (**30b**) (BSO *n* = 0.306) symmetry and di-N,N-dimethylamino-o-carborane (**31**) (BSO *n* = 0.209). However, whereas **31** has the weakest C−C bond, **30a** has the longest C−C bond at a value of 1.935 Å compared to the C−C length of 1.930 Å for **31**, confirming previous findings that the longer bond is not always the weaker bond.
2. A gap beyond 0.1 Å is present between the two longest C−C bonds (**30a** and **31**) and the other CC bonds within this study. This gap is due to the C−C bonds within molecules **30a** and **31** being governed by electronic effects. The C−C bond of **30a** is heavily affected by its electron deficient nature while the CC bond length of system **31** is influenced by negative hyperconjugation effects.
3. The covalent character of all C−C bonds has been verified via the negative energy density $H_{b}$ values taken at the bond critical point **r${}_{b}$**. In most cases $H_{b}$ is in line with the $k^{a}$ or BSO *n* values, reflecting that stronger bonds have more covalent character. However, we also found some exceptions such as the chlorinated ethanes (**28** and **29**). The results demonstrate that the local mode force constant is a sensitive bond strength measure that considers subtle second order effects that are not considered for the energy density evaluated just at the bond critical point which describes the covalent character, whereas $k^{a}$ includes counterbalancing steric repulsion effects or C−C bond weakening via delocalization of charge from the Cl lone pair into the $\sigma^{*}$(C−C), i.e., the so-called negative hyperconjugation effect, or intramolecular halogen-halogen dispersion interaction.
4. Although there is some trend that the BDH value increases with increasing C−C bond strength, the overall correlation is moderate (R${}^{2}$ = 0.893), revealing that the BDH is an inadequate measure for the intrinsic strength of the CC bond as BDH includes the overall effects of bond breakage. Such concerns in particular crowded molecules with greater possibilities for geometry and electron density reorganization of the fragments such as molecules **7**, **13**, and **15**.
5. We found an interesting relationship between the %s-character and the strength of the C−C bond as expressed via the local mode force constant. As the average %s-character of the targeted C−C bond increases the C−C bond strength increases which adds to the %s-character model quantity a physical relevance.

Overall, this study shows that the local stretching vibrational force constant is the perfect tool for comprehending the nature of CC bond elongation which, as shown in this work, is often a result of a complex interplay of steric/strain and electronic effects that sometimes add to the weakening but also may work in a counterbalancing fashion. The local mode force constants can quickly be calculated after a routine frequency calculation and therefore, can provide important guidance for the synthesis of a compound with a C−C bond of a particular strength. Work is in progress to investigate long CC bonds in nanotubes and crystals under pressure at ambient temperature [139,140,141,142,143].

## Figures and Tables

**Table 1:** Summary of geometry, calculated bond distances ($R_{calc}$), experimental bond distances ($R_{exp}$), calculated bond dissociation enthalpies (BDH${}_{calc}$), experimental calculated bond dissociation enthalpies (BDH${}_{exp}$), vibrational spectroscopy data, electron densities ($\rho_{b}$), and energy densities ($H_{b}$) of targeted CC bonds for molecules **1**–**53** ${}^{a}$.

#	sym	bond	$R_{calc}$	$R_{exp}$	BDH${}_{calc}$	BDH${}_{exp}$	$k^{a}$	$\omega^{a}$	BSO *n*	$\rho_{b}$	$H_{b}$
1	D_3d_	C−C	1.523	1.536 [97]	89.0	89.7 [98]	4.216	1092	1.000	1.659	−1.431
2	C_2v_	C−C	1.524	1.528 [99]	87.4	87.2 [98]	4.160	1085	0.989	1.671	−1.443
3	C_3v_	C−C	1.526	1.535 [100]	86.7	88.9 [98]	4.092	1076	0.976	1.675	−1.442
4	T	C−C	1.530	1.539 [26]	87.5	86.0 [98]	3.997	1063	0.958	1.669	−1.423
5	C_2h_	C−C	1.541	1.544 [25]	82.8	86.6 [98]	3.786	1035	0.917	1.645	−1.369
6	D_3_	C−C	1.577	1.582 [25]	80.4	76.0 [98]	3.229	956	0.807	1.544	−1.181
7	C_2_	C−C	1.542	1.550 [101]	66.9	66.6 [98]	3.675	1020	0.895	1.601	−1.322
8	C_2_	C−C	1.629	1.677 [18]	53.4	43.7 [102]	2.414	826	0.639	1.387	−1.925
9	C_1_	C−C	1.597	1.601 [103]	73.1	60.2 [104]	2.888	904	0.737	1.481	−1.079
10	C_2_	C−C	1.611	1.635 [103]	61.2	51.0 [104]	2.693	873	0.697	1.443	−1.016
11	C_2_	C−C	1.631	1.635 [105]	52.2	44.7 [105]	2.290	805	0.611	1.371	−1.918
12	D_2_	C−C	1.699	-	16.6	-	1.518	678	0.439	1.188	−1.663
13	S_6_	C−C	1.669	1.670 [106]	33.9	-	1.919	737	0.526	1.275	−1.830
14	C_2h_	C−C	1.523	1.539 [26]	86.0	87.2 [98]	4.177	1087	0.993	1.672	−1.447
15	C_3v_	C−CN	1.472	1.460 [107]	119.2	115.8 [98]	4.432	1120	1.041	1.787	−1.852
		C−C	1.535	-	-	-	3.915	1052	0.942	1.644	−1.388
16	C_2_	C−C	1.599	1.601 [103]	70.3	62.2 [106]	2.853	898	0.730	1.477	−1.070
17	C_2_	C−C	1.622	1.630 [18]	50.0	44.0 [106]	2.509	842	0.658	1.410	−1.965
18	C_2_	C−C	1.589	1.606 [103]	72.1	57.8 [106]	3.050	929	0.770	1.506	−1.119
19	C_1_	C−C	1.614	1.660 [19]	78.6	-	2.792	889	0.717	1.427	−1.996
20	C_1_	C−C	1.647	1.647 [18]	71.4	≈ 71 [18]	2.401	824	0.635	1.331	−1.855
21	C_1_	C−C	1.656	1.659 [18]	64.3	-	2.245	797	0.602	1.310	−1.824
22	C_1_	C−C	1.693	1.704 [18]	-	-	1.874	728	0.521	1.218	−1.694
23	C_1_	C−C	1.787	-	-	-	1.142	568	0.351	1.014	−1.465
24	C_1_	C−C	1.695	1.707 [18]	-	-	1.861	726	0.519	1.211	−1.687
25	D_2h_	C−C	1.642	1.640 [108]	-	-	2.411	826	0.637	1.317	−1.857
26	C_1_	C−C	1.708	1.754 [31]	-	-	1.788	711	0.502	1.153	−1.620
27	C_2h_	C−C	1.651	-	-	-	1.591	671	0.456	1.347	−1.876
28	C_3v_	C−C	1.512	1.516 [109]	86.9	88.3 [98]	4.154	1084	0.988	1.747	−1.615
29	D_3d_	C−C	1.583	1.564 [110]	73.4	70.1 [98]	2.944	913	0.749	1.575	−1.181
30a	D_3d_	C−C	1.935	-	41.2	-	0.894	503	0.286	0.523	−1.194
30b	C_2h_	C−C	1.591	-	42.7	-	0.971	524	0.306	1.272	−1.929
31	C_2v_	C−C	1.930	-	-	-	0.604	413	0.209	0.742	−1.242
32	C_s_	C−C	1.495	1.501 [99]	100.0	100.9 [98]	4.575	1138	1.068	1.770	−1.637
		C=C	1.324	1.336 [99]	-	-	9.821	1667	1.997	2.444	−1.197
33	D_2h_	C=C	1.322	1.339 [97]	173.9	172.2 [98]	9.961	1679	2.000	2.449	−1.214
34	C_2h_	C−C	1.457	1.467 [111]	-	116.0 [98]	5.119	1203	1.169	1.920	−1.934
		C=C	1.329	1.349 [111]	-	-	9.537	1642	1.931	2.426	−1.145
35	C_1_	C−C	1.497	1.502 [112]	98.2	99.6 [98]	4.484	1126	1.051	1.776	−1.639
		C=C	1.324	1.340 [112]	-	-	9.804	1665	1.975	2.443	−1.195
36	C_s_	C−C	1.501	1.500 [113]	97.3	99.7 [98]	4.382	1113	1.032	1.775	−1.628
		C=C	1.324	1.341 [113]	-	-	9.811	1666	1.976	2.443	−1.198
37	C_s_	C−C	1.520	1.522 [103]	96.1	97.5 [98]	4.179	1087	0.993	1.749	−1.573
		C=C	1.324	-	-	-	9.765	1662	1.968	2.438	−1.188
38	D_*∞*h_	C≡C	1.194	1.208 [97]	228.1	229.9 [98]	17.777	2243	3.190	2.894	−1.700
39	C_3v_	C−C	1.455	1.450 [26]	124.3	123.5 [98]	5.254	1219	1.194	1.844	−1.895
		C≡C	1.196	1.207 [26]	-	-	17.515	2226	3.153	2.862	−1.742
40	C_3v_	C−C	1.455	1.458 [99]	122.9	121.1 [98]	5.141	1206	1.173	1.828	−1.931
41	C_s_	C−C	1.425	1.431 [26]	135.3	133.6 [98]	5.777	1278	1.289	1.974	−1.151
		C=C	1.329	-	-	-	9.564	1645	1.936	2.418	−1.137
		C≡C	1.199	-	-	-	17.264	2210	3.116	2.860	−1.699
42	C_s_	C−C	1.430	1.429 [114]	131.7	132.1 [98]	5.582	1256	1.254	1.938	−1.141
		C=C	1.327	1.339 [114]	-	-	9.645	1656	1.957	2.428	−1.166
43	D_*∞*h_	C−C	1.372	1.383 [26]	158.3	155.0 [98]	7.406	1447	1.575	2.142	−1.517
		C≡C	1.199	1.209 [115]	-	-	17.160	2203	3.101	2.858	−1.684
44	C_*∞*v_	C−C	1.375	1.379 [116]	150.9	152.4 [98]	7.348	1442	1.565	2.122	−1.491
		C≡C	1.196	1.204 [116]	-	-	17.470	2223	3.146	2.878	−1.708
45	C_s_	C−C	1.504	1.512 [117]	103.4	103.9 [98]	4.528	1132	1.059	1.745	−1.581
46	C_s_	C−C	1.506	1.524 [118]	101.8	102.3 [98]	4.446	1121	1.044	1.751	−1.585
47	C_s_	C−C	1.514	1.500 [119]	125.0	102.1 [98]	4.274	1100	1.011	1.732	−1.540
48	C_s_	C−C	1.530	1.524 [120]	99.4	97.4 [98]	3.966	1059	0.952	1.681	−1.439
49	C_s_	C−C	1.486	1.520 [121]	-	111.9 [122]	4.751	1159	1.101	1.815	−1.708

50	C_s_	C−C	1.471	1.475 [123]	-	116.9 [98]	4.919	1180	1.132	1.868	−1.820
		C=C	1.327	-	-	-	9.549	1644	1.933	2.427	−1.154
51	C_2v_	C−C	1.430	1.436 [124]	-	140.7 [98]	5.750	1275	1.284	1.961	−1.112
		C≡C	1.198	-	-	-	17.286	2211	3.119	2.860	−1.707
52	C_2v_	C−C	1.433	1.438 [124]	-	132.7 [98]	5.569	1255	1.252	1.929	−1.116
53	D_2_	C−C	1.482	1.480 [125]	-	118.0 [98]	4.850	1171	1.120	1.840	−1.752


^*a*^ Calculated and experimentally determined CC bond distances R(CC) in Å. CC local stretching force constants (*k^a^*) in mdyn/Å, local vibrational mode frequencies (ω^*a*^) in cm^−1^, and bond strength order represented by BSO *n* values. Calculated and experimentally determined bond dissociation enthalpies (BDH) in kcal/mol. More detailed information on the experimental values is provided in the Supporting Information, Tables S1 and S2. The electron density at the CC bond critical point **r**_*b*_ in *e*/Å^3^ and the energy density at the CC bond critical point H_*b*_ in *Hartree*/Å^3^. Calculated BDH values were computed with G4 and all other values were calculated with ωB97X-D/aug-cc-pVTZ. “-” no data available. The numbers within the table correspond to the molecules shown in Figure 1.

**Table 2:** % s-character of the C atoms C1 and C2 forming the targeted C−C bond in Group VI and Group VII molecules determined from the NBO analysis ${}^{a}$.

#	% s (C1)	% s (C2)	Sum of % s Character	Av. of % s Character
**1**	29.6 (sp${}^{3}$)	29.6 (sp${}^{3}$)	59.2	29.6
**33**	40.7 (sp${}^{2}$)	40.7 (sp${}^{2}$)	81.4	40.7
**38**	52.3 (sp)	52.3 (sp)	104.6	52.3
**32**	30.2 (sp${}^{3}$)	33.2 (sp${}^{2}$)	63.4	31.7
**34**	33.7 (sp${}^{2}$)	33.7 (sp${}^{2}$)	67.4	33.7
**35**	28.8 (sp${}^{3}$)	33.4 (sp${}^{2}$)	62.2	31.1
**36**	27.2 (sp${}^{3}$)	33.7 (sp${}^{2}$)	60.8	30.4
**37**	25.6 (sp${}^{3}$)	34.4 (sp${}^{2}$)	60.0	30.0
**39**	29.5 (sp${}^{3}$)	47.7 (sp)	77.1	38.6
**40**	26.8 (sp${}^{3}$)	52.4 (sp)	79.3	39.7
**41**	47.9 (sp)	32.5 (sp${}^{2}$)	80.4	40.2
**42**	52.2 (sp)	30.3 (sp${}^{2}$)	82.5	41.3
**43**	46.9 (sp)	46.9 (sp)	93.8	46.9
**44**	45.7 (sp)	50.7 (sp)	96.4	48.2
**45**	31.1 (sp${}^{2}$)	30.3 (sp${}^{3}$)	61.4	30.7
**46**	31.5 (sp${}^{2}$)	30.0 (sp${}^{3}$)	61.5	30.8
**47**	31.9 (sp${}^{2}$)	27.5 (sp${}^{3}$)	59.4	29.7
**48**	32.5 (sp${}^{2}$)	26.0 (sp${}^{3}$)	58.5	29.3
**49**	31.7 (sp${}^{2}$)	32.3 (sp${}^{3}$)	64.0	32.0
**50**	32.0 (sp${}^{2}$)	34.0 (sp${}^{2}$)	66.0	33.0
**51**	30.6 (sp${}^{2}$)	48.0 (sp)	78.7	39.4
**52**	28.6 (sp${}^{2}$)	52.5 (sp)	81.1	40.6
**53**	32.0 (sp${}^{2}$)	32.0 (sp${}^{2}$)	64.0	32.0

${}^{a}$ C1 and C2 are numbered according to Figure 1, e.g., in **39** C1 is the methyl carbon and C2 is the alkyne carbon atom. The hybridization of the carbon atom is given in parenthesis. The s-character percentages are based upon ωB97X-D/aug-cc-pVTZ calculations.

## Data Availability

The data presented in this study are available in Supplementary Materials.

## Supplementary Materials

The following are available online, Figure S1: Scheme of molecules **1-53** with atomic charges from the natural bond orbital (NBO) analysis. Table S1: Provides a summarized compilation of experimental bond lengths of the targeted compounds, references are listed. Table S2: Lists experimental bond dissociation enthalpies (BDH) values, references are cited. Table S3: Provides IUPAC nomenclature for systems. Table S4: Summarizes the calculated bond distances R(CC) in Å, local mode force constants k${}^{a}$ (CC) in mdyn/Å, energy densities H${}_{b}$ at the bond critical point r${}_{b}$ in Hartree/Å${}^{3}$ and NBO charges in electrons for the CC bonds of fluorinated and chlorinated ethane at different levels of theory. The optimized coordinates of molecules **1**–**53**, obtained at the ωB97X-D/aug-cc-pVTZ level of theory, are provided at the end of the SI.
