# Supplementary material for: Exceptionally Long Covalent CC Bonds—A Local Vibrational Mode Study
Source: Molecules. 2021 Feb 11;26(4):950. doi: 10.3390/molecules26040950 (PMC7916873; doi:10.3390/molecules26040950)
Supplement: Supplementary file 1 [file molecules-26-00950-s001.pdf]

Supporting Material for:

Exceptionally Long CC Covalent Bonds - A Local Vibrational Mode Study

Alexis A. A. Delgado,[1] Alan Humason,[1] Kalescky,[1] and Elfi Kraka[1, a)]

Department of Chemistry, Computational and Theoretical Chemistry Group (CATCO), Southern Methodist University, 3215 Daniel Avenue, Dallas, TX 75275-0314, United States

(Dated: 19 February 2021)

[a)]Electronic mail: ekraka@gmail.com

I. NOTES

The compilation of experimental bond lengths summarized in Table S1, is from an extensive literature search of the target compounds.[1–42] This data includes electron diffraction, x-ray diffraction, microwave spectroscopy and infrared spectroscopy methods. The parameters reported in the literature are obtained by different techniques, at different temperatures and in different matrices, all of which effects the actual values. Note that when multiple values are available, the experimental values vary by an average of 0.034 Å.

To simplify the on-going analysis, the most accurate bond lengths are selected from among the literature values by the following criteria:

- Gas phase electron diffraction experiments, yielding r_a values, are considered most accurate, because they are taken in the gas phase, making them more comparable to the theoretical results. These results are also more accurate than x-ray diffraction experiments, because the wavelengths of accelerated electrons are shorter than the wavelengths of x-rays, further increasing the accuracy.[43] In some experiments, the electron diffraction results are combined with microwave spectroscopy rotational constants, to obtain r_g values of improved accuracy.

- X-ray diffraction experiments generally have the limitations that 1) structures are determined on solid crystals, and therefore are subject to packing effects, and 2) hydrogen atoms, which have no core electrons, cannot be accurately located. For this study of C-C bond lengths, the approximate hydrogen locations are not a concern, but the other considerations make these values less reliable than electron diffraction.

- Microwave and infrared spectroscopy experiments, yielding r_m, r_o and r_s data, depend upon comparison of rotational constants for multiple isotopomers. These methods have the limitations that 1) they are dependent upon the availability of the isotopomers, and 2) the calculations are based on initial assumed geometries. These results are considered least accurate, and are used when results from the other techniques are unavailable.

Although bond dissociation energies (BDE's) are often sited as bond strength descriptors, molecular energies at 0K cannot be measured. Experimentally, only bond dissociation enthalpies

(BDH's) are accessible. Therefore, calculated (G4) values of BDH at 298K are used for comparison with reported experimental values. Several compilations of bond dissociation enthalpies are available,[44–49] and are compiled in Table S2. Here again, the available data are from various methods, and are not comparable. Where multiple values are available, the BDH's vary by an average of 4 kcal/mol. We make no differentiation of data quality here. IUPAC names for all of the target compounds are included in Table S3, to remove any ambiguity concerning structure. Calculated bond distances R(CC) , local mode force constants k^a(CC), energy densities, and natural bond orbital (NBO) charges in electrons for the CC bonds of fluorinated and chlorinated ethane at different levels of theory are provided in Table S4. Figure S1 features a NBO charge schematic for molecules **1-53** calculated at the ωB97X-D/aug-cc-pVTZ level of theory. Figure S2 demonstrates the relationship between calculated bond dissociation enthalpies BDH_{calc} and calculated C−C bond lengths. BDH_{calc} values were obtained with the composite G4 method and R(C−C) values computed at the ωB97X-D/aug-cc-pVTZ level of theory. Figure S3 shows the correlation between the local stretching force constant k^a and electron density ρ_b calculated at the ωB97X-D/aug-cc-pVTZ level of theory.

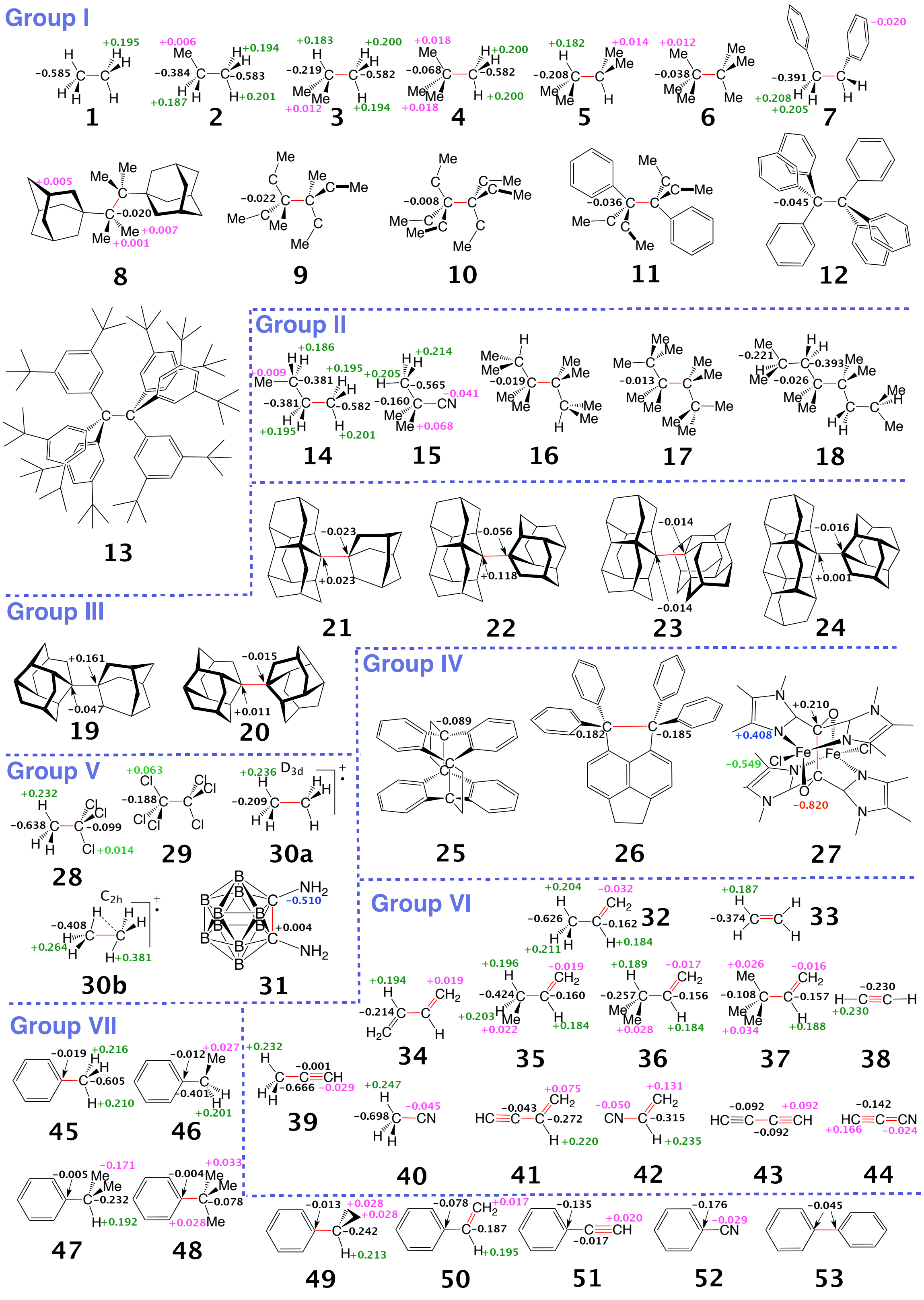

FIG. S1. Natural bond orbital (NBO) charges in e for molecules **1**-**53** calculated at the ωB97X-D/aug-cc-pVTZ level of theory.

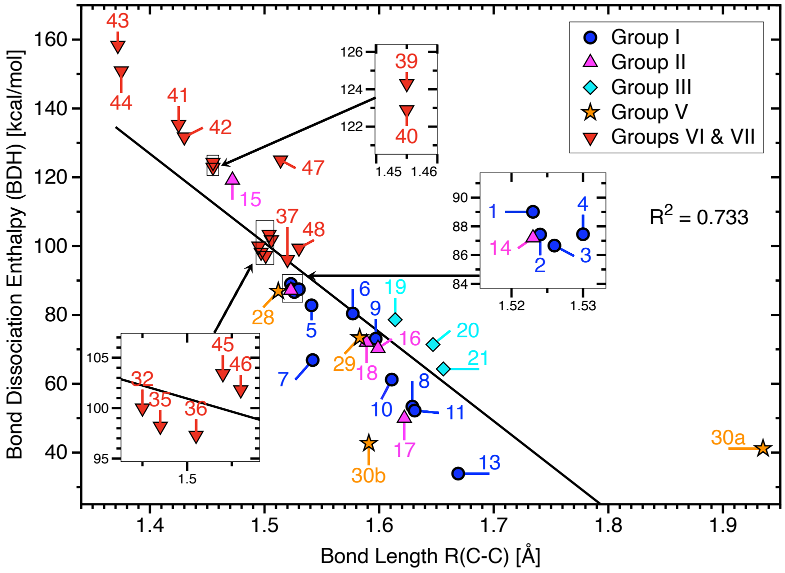

FIG. S2. Correlation between calculated bond dissociation enthalpies BDH_{calc} and calculated C−C bond lengths. BDH values calculated with the composite G4 method and R(C−C) values at the ωB97X-D/aug-cc-pVTZ level of theory. The black line denotes a linear relationship.

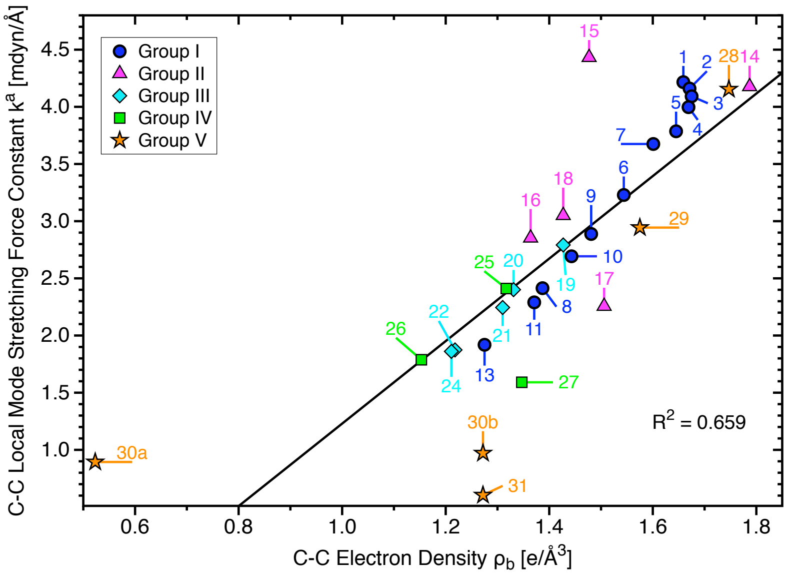

FIG. S3. Correlation between the local stretching force constant k^a and electron density ρ_b calculated at the ωB97X-D/aug-cc-pVTZ level of theory. The black line denotes a linear relationship.

TABLE S1. Experimental Carbon-Carbon Bond Lengths R [Å]

No.	Bond ID	Electron Diffraction	X-Ray Diffraction	Microwave Spectroscopy	Infrared Spectroscopy	ΔR^d_{exp}
1	Me-Me	**1.536**[43]	1.536[32]		1.534[43]	0.002
2	Me-Et			**1.528**[38]		
3	iPr-Me	**1.535** (1)[2]		1.525 (1)[3]		0.01
4	tBu-Me	**1.539**[43]	1.546(2)[11]			0.007
5	$(iPr)_2$	**1.544**[39]				
6	$(tBu)_2$	**1.582**[39]				
7	$(PhCH_2)_2$	**1.55 (26)**[25]	1.529 (3)[25]			
			1.58[48]			0.051
8	$(AdMe_2C)_2$	**1.677 (30)**[40]	1.639[26]			0.038
9	$(Et_2MeC)_2$	**1.601**[48]				
10	$(Et_3C)_2$	1.635[48]				
11	$(PhEt_2C)_2$		**1.635**[27]			
12	$(Ph_3C)_2$					
13	hexakis		**1.67 (3)**[42]			
14	Et-Et	**1.539** (3)[43]				
15	tBu-C≡N	**1.46 (2)**[14]				
16	$(iPrMe_2C)_2$	1.601[48]				
17	$(tBuMe_2C)_2$	**1.63**[40]				
18	$(iBuMe_2C)_2$	1.606[48]				
19	diAd-Ad		**1.66**[41]			
20	diAd-diAd		**1.647**[40]			
21	triAd-Ad		**1.659**[40]			
22	triAd-diAd		**1.704**[40]			
23	triAd-triAd					
24	tetraAd-diAd		**1.707**[40]			
25	bianthracene	**1.64 (1)**[28]	1.653[29]			
			1.648 (3)[29]			
			1.77[30]			0.13
26	tetra-Ph-acenaphthene		**1.754 (2)**[31]			
28	H_3C-CCl_3			**1.516** (4)[12]		
29	$(Cl_3C)_2$	**1.564** (14)[13]				
30	CH_3-CH_3^+ D_{3d}					
31	CH_3-CH_3^+ C_{2h}					
32	Me-CH=CH_2			**1.501**[38]		
33	H_2C=CH_2		**1.339**[32]			
34	$(CH_2$=$CH)_2$	**1.467**[5]				
		1.486[43]			1.485[43]	0.018
35	Et-CH=CH_2	**1.502**[4]				
36	iPr-CH=CH_2				**1.500**[10]	
37	tBu-CH=CH_2	**1.522**[48]				
38	HC≡CH		**1.208**[32]			
39	Me-C≡CH	**1.45**[43]		1.458[38]	1.459[43]	0.009
				1.4589[1]		
				1.4577[1]		
40	Me-C≡N			**1.458**[38]		
				1.45836[1]		
				1.4582[1]		0.004
41	CH_2=CH-C≡CH	**1.4314**[43]			1.426[43]	0.005
42	CH_2=CH-C≡N			**1.429** (5)[6]		
				1.4430 (2)[6]	1.430[7]	
				1.458[38]	1.429[7]	0.029
43	HC≡C-C≡CH	**1.383**[43]		1.3741[9]		0.011
44	HC≡C-C≡N			**1.3796**[8]		
				1.3793[9]		
				1.206[38]		
				1.3775[1]		
				1.382[1]		0.176
45	Me-Ph	**1.5214 (65)**[15]		1.512 (1)[33]		
		1.523[15]				
		1.51 (2)[16]				0.013
46	Et-Ph	**1.524 (9)**[17]				
47	iPr-Ph	**1.50 (5)**[21]				
48	tBu-Ph	**1.5237**[23]				
49	Ph-cycloC_3H_5	**1.520 (25)**[22]				
50	Ph-CH=CH_2	**1.475 (23)**[18]				
51	Ph-C≡CH	**1.436 (4)**[19]		1.4447 (7)[19]		0.009
52	Ph-C≡N	**1.438 (5)**[19]		1.444[19]		0.006
		1.434 (3)[19]		1.4509 (6)[19]		
				1.388[38]		
				1.4509 (6)[20]		0.063
53	Ph-Ph		1.48[24]			

TABLE S2. Experimental Bond Dissociation Enthalpies [kcal/mol].

No.	Bond ID	NIST[46]	Luo[44]	Griller[49]	Other Refs	ΔBDH_{exp}
1	Me-Me	89.68	90.2 ± 0.2	88.8	88±2[43]	2.2
2	Me-Et	87.2	88.5 ± 0.5	87.4		1.3
3	iPr-Me	88.9	88.2 ± 0.9	85.7	87±2[43]	3.2
4	tBu-Me	86	86.9 ± 0.7	84	82[43]	4.9
5	(iPr)2	86.6	84.5 ± 1.1	81		5.6
6	(Me3C)2 (tBu)2	76	68.3 ± 1.5	72.2		4.9
7	(PhCH2)2	66.6	62.6 ± 2.2			4
8	(AdMe2C)2				43.7[26]	
9	(Et2MeC)2				60.2[37]	
10	(Et3C)2				51.0[37]	
11	(PhEt2C)2				44.7[27]	
14	Et-Et	87.2	86.8 ± 0.6	86		1.2
15	tBu-C≡N	115.8	117.7 ± 1.3	109.2		8.5
16	(iPrMe2C)2				62.2[37]	
17	(tBuMe2C)2				44.0[37]	
18	(iBuMe2C)2				57.8[37]	
21	H3C-CCl3	88.3	87.6 ± 2.0		87.6 ± 2.0[36]	0.7
29	(Cl3C)2	70.1	68.3 ± 1.5		70.1 ± 3.5[50]	1.8
32	Me-CH=CH2	100.9		93		7.9
33	H2C=CH2	172.2	174.1 ± 1.5			2.1
34	(CH2=CH)2	116	116.9 ± 1.5			0.9
35	Et-CH=CH2	99.6	100.0 ± 1.0	91.4		8.6
36	iPr-CH=CH2	99.7	99.2 ± 1.5	89.2		10.5
37	tBu-CH=CH2	97.5	97.7 ± 1.3	87.3		10.4
38	HC≡CH	229.9	229.5 ± 1.0			0.4
39	Me-C≡CH	123.5	126.0 ± 1.0	122.2		3.8
40	Me-C≡N	121.1	124.7	118.2	122.8[34]	6.4
41	CH2=CH-C≡CH	133.6				
42	CH2=CH-C≡N	132.1	133.9 ± 1.8			1.8
43	HC≡C-C≡CH	155				
44	HC≡C-C≡N	152.4	143.9			8.5
45	Me-Ph	103.9	102.0 ± 1.0	101		2.9
46	Et-Ph	102.3	100.2 ± 1.0	99.5		2.8
47	iPr-Ph	102.1	98.9 ± 1.2	96.8		5.3
48	tBu-Ph	97.4		93.4		4
49	Ph-cycloC3H5		109.8 ± 1.2		111.9[47]	2.1
50	Ph-CH=CH2	116.9	115.2 ± 1.3			1.7
51	Ph-C≡CH	140.7			141.2 ± 1.4[35]	0.5
52	Ph-C≡N	132.7	132.8 ± 2.0			0.1
53	Ph-Ph	118	114.4 ± 1.5			3.6

TABLE S3. IUPAC Name for Compounds **1** - **53**

No.	Bond ID	IUPAC Name
1	Me-Me	ethane
2	Me-Et	propane
3	iPr-Me	2-methylpropane
4	tBu-Me	2,2-dimethylpropane
5	$(iPr)_2$	2,3-dimethylbutane
6	$(tBu)_2$	2,2,3,3-tetramethylbutane
7	$(PhCH_2)_2$	1,2-diphenylethane
8	$(AdMe_2C)_2$	2,3-diadamantyl-2,3-dimethylbutane
9	$(Et_2MeC)_2$	3,4-ethyl-3,4-methylhexane
10	$(Et_3C)_2$	3,3,4,4-tetraethylhexane
11	$(PhEt_2C)_2$	3,4-ethyl-3,4-phenylhexane
12	$(Ph_3C)_2$	hexaphenylethane
13	hexakis	hexakis-(3,5-di-tert-butylphenyl)ethane
14	Et-Et	butane
15	tBu-C≡N	2-cyano-2-methylpropane
16	$(iPrMe_2C)_2$	2,3,3,4,4,5-hexamethylhexane
17	$(tBuMe_2C)_2$	2,2,3,3,4,4,5,5-octamethylhexane
18	$(iBuMe_2C)_2$	2,4,4,5,5,7-hexamethyloctane
19	diAd-Ad	1-(1-adamantyl)diamantane
20	diAd-diAd	1-(1-diamantyl)diamantane
21	triAd-Ad	2-(1-adamantyl)triamantane
22	triAd-diAd	2-(1-diamantyl)triamantane
23	triAd-triAd	2-(2-triamantyl)triamantane
24	tetraAd-diAd	2-(1-diamantyl)[121]tetramantane
25	bianthracene	bi(anthracene-9,10-dimethylene)
26	tetra-Ph-acenaphthene	acenaphthene-5,6-diyl bis(diphenylmethylium)
27	iron clamp	bis(1,4,5-trimethylimidazolyl)ketone iron complex
28	$H_3C\text{-}CCl_3$	1,1,1-trichloroethane
29	$(Cl_3C)_2$	hexachloroethane
30a	$CH_3CH_3^+$ D_{3d}	ethane cation, D_{3d}
30b	$CH_3CH_3^+$ C_{2h}	ethane cation, C_{2h}
31	C-C	di-N,N-dimethylamino-o-carborane
32	$Me\text{-}CH{=}CH_2$	propene
33	$H_2C{=}CH_2$	ethene
34	$(CH_2{=}CH)_2$	1,3-butadiene
35	$Et\text{-}CH{=}CH_2$	1-butene
36	$iPr\text{-}CH{=}CH_2$	3-methyl-1-butene
37	$tBu\text{-}CH{=}CH_2$	3,3-dimethyl-1-butene
38	HC≡CH	ethyne
39	Me-C≡CH	propyne
40	Me-C≡N	acetonitrile
41	$CH_2{=}CH\text{-}C{\equiv}CH$	buta-1-en-3-yne
42	$CH_2{=}CH\text{-}C{\equiv}N$	cyanoethene
43	HC≡C-C≡CH	1,3-butadiyne
44	HC≡C-C≡N	cyanoethyne
45	Me-Ph	toluene
46	Et-Ph	ethylbenzene
47	iPr-Ph	cumene
48	tBu-Ph	(1,1-dimethylethyl)benzene
49	$Ph\text{-}cycloC_3H_5$	cyclopropylbenzene
50	$Ph\text{-}CH{=}CH_2$	styrene
51	Ph-C≡CH	phenylacetylene
52	Ph-C≡N	cyanobenzene
53	Ph-Ph	biphenyl

TABLE S4. Calculated bond distances R(CC) in Å, local mode force constants k^a (CC) in mdyn/Å, energy densities H_b at the bond critical point r_b in Hartree/$Å^3$ and NBO charges in electrons for the CC bonds of fluorinated and chlorinated ethane at different levels of theory.

**Molecule**	**R(CC)**	**k^a**	**H$_b$**	**Charge C1**	**Charge C2**
***ω*B97X-D/aug-cc-pVTZ**					
H_3C-CH_3	1.523	4.216	-1.431	-0.586	-0.586
H_3C-CH_2Cl	1.511	4.310	-1.548	-0.591	-0.441
$H_3C-CHCl_2$	1.507	4.282	-1.607	-0.590	-0.359
H_3C-CCl_3	1.512	4.154	-1.615	-0.588	-0.313
CCl_3-CH_2Cl	1.526	3.863	-1.511	-0.317	-0.455
CCl_3-CHCl_2	1.550	3.424	-1.361	-0.319	-0.378
ClC_3-CCl_3	1.583	2.944	-1.181	-0.328	-0.328
H_3C-CH_2F	1.508	4.365	-1.608	-0.643	+0.085
H_3C-CHF_2	1.501	4.454	-1.732	-0.694	+0.630
H_3C-CF_3	1.501	4.510	-1.792	-0.737	+1.089
CF_3-CH_2F	1.519	4.239	-1.818	+1.033	-0.069
CF_3-CHF_2	1.540	4.010	-1.627	+0.985	+0.477
F_3C-CF_3	1.555	3.943	-1.560	+0.962	+0.962
**PBE0-D3/aug-cc-pVTZ**					
H_3C-CH_3	1.520	4.286	-1.456	-0.601	-1.601
H_3C-CH_2Cl	1.507	4.378	-1.573	-0.622	-0.348
$H_3C-CHCl_2$	1.503	4.355	-1.636	-0.637	-0.194
H_3C-CCl_3	1.507	4.232	-1.649	-0.655	-0.112
CCl_3-CH_2Cl	1.522	3.909	-1.540	-0.147	-0.431
CCl_3-CHCl_2	1.547	3.447	-1.384	-0.174	-0.287
ClC_3-CCl_3	1.580	2.926	-1.201	-0.206	-0.206
H_3C-CH_2F	1.504	4.435	-1.632	-0.644	+0.069
H_3C-CHF_2	1.497	4.530	-1.758	-0.675	+0.589
H_3C-CF_3	1.497	4.585	-1.821	-0.699	+1.013
CF_3-CH_2F	1.515	4.310	-1.748	+0.970	-0.026
CF_3-CHF_2	1.536	4.079	-1.656	+0.943	+0.501
F_3C-CF_3	1.550	4.015	-1.589	+0.922	+0.922

Optimized coordinates of all molecules in this study

Molecule 1

C	0.00000	0.00000	0.76156
C	0.00000	0.00000	-0.76156
H	-0.22142	0.99159	1.15708
H	0.22142	-0.99159	-1.15708
H	0.96945	-0.30404	1.15708
H	-0.74803	-0.68755	1.15708
H	0.74803	0.68755	-1.15708
H	-0.96945	0.30404	-1.15708

Molecule 2

C	0.00000	1.26755	-0.25924
C	0.00000	0.00000	0.58627
C	0.00000	-1.26755	-0.25924
H	0.00000	2.16395	0.36127
H	-0.88054	1.30657	-0.90333
H	0.88054	1.30657	-0.90333
H	-0.87362	0.00000	1.24202
H	0.87362	0.00000	1.24202
H	0.00000	-2.16395	0.36127
H	0.88054	-1.30657	-0.90333
H	-0.88054	-1.30657	-0.90333

Molecule 3

C	0.00000	0.00000	0.76156
C	0.00000	0.00000	-0.76156

H	-0.22142	0.99159	1.15708
H	0.22142	-0.99159	-1.15708
H	0.96945	-0.30404	1.15708
H	-0.74803	-0.68755	1.15708
H	0.74803	0.68755	-1.15708
H	-0.96945	0.30404	-1.15708

Molecule 4

C	-0.77362	-1.24560	0.43739
C	0.00000	0.00000	0.00000
C	0.47540	0.76553	1.23663
C	1.21043	-0.41912	-0.83697
C	-0.91221	0.89918	-0.83705
H	1.03151	1.66092	0.95226
H	-0.37037	1.07695	1.85269
H	1.12944	0.14547	1.85275
H	1.78120	0.45263	-1.16273
H	1.87912	-1.06282	-0.26224
H	0.89867	-0.96839	-1.72745
H	-0.38380	1.79724	-1.16280
H	-1.26632	0.37622	-1.72753
H	-1.78567	1.21326	-0.26237
H	-1.64431	-0.97432	1.03750
H	-0.14451	-1.90580	1.03756
H	-1.12496	-1.81136	-0.42766

Molecule 5

C	-0.16489	0.75261	0.00000
C	0.16489	-0.75261	0.00000
H	1.25786	-0.84305	0.00000

C	-0.36571	-1.45872	1.24747
C	-0.36571	-1.45872	-1.24747
H	-0.17398	-2.53090	1.19261
H	0.09675	-1.09014	2.16160
H	-1.44635	-1.31961	1.33671
H	-0.17398	-2.53090	-1.19261
H	-1.44635	-1.31961	-1.33671
H	0.09675	-1.09014	-2.16160
H	-1.25786	0.84305	0.00000
C	0.36571	1.45872	1.24747
C	0.36571	1.45872	-1.24747
H	0.17398	2.53090	1.19261
H	-0.09675	1.09014	2.16160
H	1.44635	1.31961	1.33671
H	0.17398	2.53090	-1.19261
H	1.44635	1.31961	-1.33671
H	-0.09675	1.09014	-2.16160

Molecule 6

C	-0.77362	-1.24560	0.43739
C	0.00000	0.00000	0.00000
C	0.47540	0.76553	1.23663
C	1.21043	-0.41912	-0.83697
C	-0.91221	0.89918	-0.83705
H	1.03151	1.66092	0.95226
H	-0.37037	1.07695	1.85269
H	1.12944	0.14547	1.85275
H	1.78120	0.45263	-1.16273
H	1.87912	-1.06282	-0.26224
H	0.89867	-0.96839	-1.72745
H	-0.38380	1.79724	-1.16280

H	-1.26632	0.37622	-1.72753
H	-1.78567	1.21326	-0.26237
H	-1.64431	-0.97432	1.03750
H	-0.14451	-1.90580	1.03756
H	-1.12496	-1.81136	-0.42766

Molecule 7

C	0.52703	0.56262	1.95011
C	-0.52703	-0.56262	1.95011
H	0.42402	1.13471	2.87386
H	1.52189	0.11395	1.95914
H	-0.42402	-1.13471	2.87386
H	-1.52189	-0.11395	1.95914
C	0.39279	1.48162	0.76660
C	-0.52314	2.52877	0.78733
C	1.13136	1.27112	-0.39252
C	-0.69835	3.34473	-0.31902
C	0.96050	2.08411	-1.50216
C	0.04405	3.12358	-1.46950
H	-1.10579	2.70756	1.68335
H	1.83768	0.45139	-0.42953
H	-1.41264	4.15633	-0.28230
H	1.54261	1.90254	-2.39555
H	-0.08965	3.75942	-2.33401
C	-0.39279	-1.48162	0.76660
C	0.52314	-2.52877	0.78733
C	-1.13136	-1.27112	-0.39252
C	0.69835	-3.34473	-0.31902
C	-0.96050	-2.08411	-1.50216
C	-0.04405	-3.12358	-1.46950
H	1.10579	-2.70756	1.68335

H	-1.83768	-0.45139	-0.42953
H	1.41264	-4.15633	-0.28230
H	-1.54261	-1.90254	-2.39555
H	0.08965	-3.75942	-2.33401

Molecule 8

C	-0.61188	-0.53729	-0.31078
C	0.61188	0.53729	-0.31078
C	1.63377	0.19370	0.78458
C	1.34515	0.44688	-1.66722
H	2.39786	0.96483	0.85848
H	1.17468	0.09907	1.76502
H	2.15585	-0.73271	0.56961
H	2.35159	0.85344	-1.59016
H	1.44623	-0.57385	-2.00842
H	0.82734	0.99323	-2.45428
C	-1.63377	-0.19370	0.78458
C	-1.34515	-0.44688	-1.66722
H	-2.39786	-0.96483	0.85848
H	-1.17468	-0.09907	1.76502
H	-2.15585	0.73271	0.56961
H	-2.35159	-0.85344	-1.59016
H	-1.44623	0.57385	-2.00842
H	-0.82734	-0.99323	-2.45428
C	0.22985	2.11020	-0.08661
C	-0.33237	3.95774	1.60165
C	-0.05247	2.46482	1.39739
C	-0.95496	2.62901	-0.93611
C	1.43911	3.00034	-0.50782
C	-1.22375	4.12388	-0.71698
C	1.18888	4.49582	-0.28072

C	-1.53644	4.37784	0.75817
C	0.89546	4.76851	1.19284
C	-0.00139	4.94139	-1.12902
H	-0.54904	4.12533	2.65874
H	-2.08176	4.41033	-1.32869
H	2.08365	5.04230	-0.58634
H	-0.90032	1.90707	1.78032
H	0.80648	2.19605	2.01203
H	-0.74671	2.46966	-1.99659
H	-1.87214	2.09248	-0.70761
H	2.33871	2.71270	0.03757
H	1.65343	2.86477	-1.56633
H	-1.76303	5.43498	0.91958
H	-2.42037	3.80821	1.05798
H	1.75326	4.48316	1.80760
H	0.72176	5.83583	1.35356
H	-0.19198	6.00861	-0.98855
H	0.21384	4.78701	-2.18965
C	-0.22985	-2.11020	-0.08661
C	0.33237	-3.95774	1.60165
C	0.05247	-2.46482	1.39739
C	0.95496	-2.62901	-0.93611
C	-1.43911	-3.00034	-0.50782
C	1.22375	-4.12388	-0.71698
C	-1.18888	-4.49582	-0.28072
C	1.53644	-4.37784	0.75817
C	-0.89546	-4.76851	1.19284
C	0.00139	-4.94139	-1.12902
H	0.54904	-4.12533	2.65874
H	2.08176	-4.41033	-1.32869
H	-2.08365	-5.04230	-0.58634
H	0.90032	-1.90707	1.78032

H	-0.80648	-2.19605	2.01203
H	0.74671	-2.46966	-1.99659
H	1.87214	-2.09248	-0.70761
H	-2.33871	-2.71270	0.03757
H	-1.65343	-2.86477	-1.56633
H	1.76303	-5.43498	0.91958
H	2.42037	-3.80821	1.05798
H	-1.75326	-4.48316	1.80760
H	-0.72176	-5.83583	1.35356
H	0.19198	-6.00861	-0.98855
H	-0.21384	-4.78701	-2.18965

Molecule 9

C	-0.80008	0.02193	-0.17253
C	0.70191	-0.02884	0.36785
C	0.77085	0.63181	1.75630
C	1.23971	-1.47124	0.58801
C	1.65581	0.71101	-0.60223
H	1.74116	0.45411	2.21849
H	0.62463	1.70971	1.71254
H	0.01985	0.21986	2.43081
C	1.41090	-2.40366	-0.60746
H	2.21884	-1.36302	1.05756
H	0.62869	-1.96896	1.34144
C	3.10392	0.86943	-0.14576
H	1.66128	0.19260	-1.56062
H	1.25851	1.70186	-0.80965
C	-0.86978	-0.16936	-1.69618
C	-1.61510	-1.11670	0.50037
C	-1.53085	1.34983	0.16636
H	-1.90931	-0.21316	-2.02173

H	-0.40534	0.65301	-2.23625
H	-0.38484	-1.08719	-2.02012
C	-3.12949	-1.12335	0.29844
H	-1.23279	-2.07212	0.14174
H	-1.42711	-1.09950	1.57612
C	-0.98505	2.68298	-0.34072
H	-2.53573	1.25424	-0.24692
H	-1.66758	1.41582	1.24802
H	1.94630	-3.30143	-0.29643
H	0.46097	-2.72741	-1.02971
H	1.99005	-1.94264	-1.40795
H	3.67185	1.40742	-0.90512
H	3.18340	1.43693	0.78122
H	3.59778	-0.09047	0.00570
H	-3.54515	-2.04689	0.70232
H	-3.62043	-0.29765	0.81141
H	-3.40919	-1.07769	-0.75445
H	-1.72102	3.46600	-0.15398
H	-0.06451	2.98494	0.15522
H	-0.79678	2.66851	-1.41440

Molecule 10

C	0.00000	0.00000	0.78827
C	0.00000	0.00000	-0.78827
C	0.21657	-1.41442	-1.34471
C	-1.33321	0.51965	-1.34471
C	1.11664	0.89476	-1.34471
H	0.07866	-1.40859	-2.42719
H	1.22432	-1.77815	-1.14582
H	-0.49103	-2.13288	-0.92963
H	-1.25920	0.63617	-2.42719

H	-2.15209	-0.17122	-1.14582
H	-1.60161	1.49168	-0.92963
H	1.18054	0.77242	-2.42719
H	0.92776	1.94937	-1.14582
H	2.09264	0.64120	-0.92963
C	0.11542	1.42624	1.34471
C	-1.29287	-0.61316	1.34471
C	1.17745	-0.81307	1.34471
H	-0.02173	1.41061	2.42719
H	1.09475	1.86073	1.14582
H	-0.64149	2.09255	0.92963
H	-1.21077	-0.72412	2.42719
H	-2.15882	0.01771	1.14582
H	-1.49146	-1.60182	0.92963
H	1.23249	-0.68649	2.42719
H	1.06407	-1.87844	1.14582
H	2.13294	-0.49073	0.92963

Molecule 11

C	0.56915	-0.58345	-0.76479
C	-0.56915	0.58345	-0.76479
C	-0.58582	1.33872	-2.11747
C	-2.01114	0.03712	-0.52337
C	-1.58567	2.48923	-2.22051
H	0.40636	1.71136	-2.35698
H	-0.81621	0.62796	-2.90760
C	-2.74792	-0.69026	-1.64598
H	-2.01723	-0.61362	0.34660
H	-2.61864	0.89931	-0.24478
C	0.58582	-1.33872	-2.11747
C	2.01114	-0.03712	-0.52337

C	1.58567	-2.48923	-2.22051
H	-0.40636	-1.71136	-2.35698
H	0.81621	-0.62796	-2.90760
C	2.74792	0.69026	-1.64598
H	2.01723	0.61362	0.34660
H	2.61864	-0.89931	-0.24478
H	-1.46525	2.99840	-3.17700
H	-2.61578	2.13857	-2.16696
H	-1.44803	3.22579	-1.43043
H	-3.77596	-0.86998	-1.32976
H	-2.79159	-0.11373	-2.56897
H	-2.31810	-1.66036	-1.88209
H	1.46525	-2.99840	-3.17700
H	2.61578	-2.13857	-2.16696
H	1.44803	-3.22579	-1.43043
H	3.77596	0.86998	-1.32976
H	2.79159	0.11373	-2.56897
H	2.31810	1.66036	-1.88209
C	-0.26588	1.56572	0.38391
C	-0.75914	1.36224	1.67219
C	0.53203	2.69487	0.19425
C	-0.45827	2.22431	2.71611
C	0.83608	3.56270	1.22937
C	0.34430	3.33097	2.50358
H	-1.38322	0.50940	1.88686
H	0.93290	2.91571	-0.78318
H	-0.85851	2.02343	3.70092
H	1.45901	4.42547	1.03456
H	0.57818	4.00632	3.31514
C	0.26588	-1.56572	0.38391
C	0.75914	-1.36224	1.67219
C	-0.53203	-2.69487	0.19425

C	0.45827	-2.22431	2.71611
C	-0.83608	-3.56270	1.22937
C	-0.34430	-3.33097	2.50358
H	1.38322	-0.50940	1.88686
H	-0.93290	-2.91571	-0.78318
H	0.85851	-2.02343	3.70092
H	-1.45901	-4.42547	1.03456
H	-0.57818	-4.00632	3.31514

Molecule 12

C	0.00000	0.00000	0.81910
C	0.00000	0.00000	-0.81910
C	-0.30342	1.46450	1.37663
C	-0.53254	2.57039	0.54768
C	-0.36251	1.74974	2.74131
C	-0.79519	3.83812	1.03279
C	-0.62497	3.01651	3.24325
C	-0.84533	4.08014	2.39283
H	-0.51290	2.47559	-0.52001
H	-0.20652	0.99682	3.48386
H	-0.96112	4.63901	0.32534
H	-0.65380	3.15568	4.31546
H	-1.05033	5.06958	2.77749
C	0.30342	-1.46450	-1.37663
C	0.53254	-2.57039	-0.54768
C	0.36251	-1.74974	-2.74131
C	0.79519	-3.83812	-1.03279
C	0.62497	-3.01651	-3.24325
C	0.84533	-4.08014	-2.39283
H	0.51290	-2.47559	0.52001
H	0.20652	-0.99682	-3.48386
H	0.96112	-4.63901	-0.32534

H	0.65380	-3.15568	-4.31546
H	1.05033	-5.06958	-2.77749
C	-1.42000	0.46948	-1.37663
C	-2.49229	0.82400	-0.54768
C	-1.69658	0.56092	-2.74131
C	-3.72150	1.23040	-1.03279
C	-2.92486	0.96702	-3.24325
C	-3.95618	1.30799	-2.39283
H	-2.40037	0.79361	0.52001
H	-0.96653	0.31956	-3.48386
H	-4.49806	1.48715	-0.32534
H	-3.05980	1.01163	-4.31546
H	-4.91555	1.62518	-2.77749
C	1.11659	0.99502	-1.37663
C	1.95976	1.74639	-0.54768
C	1.33406	1.18882	-2.74131
C	2.92631	2.60771	-1.03279
C	2.29989	2.04949	-3.24325
C	3.11084	2.77215	-2.39283
H	1.88748	1.68198	0.52001
H	0.76001	0.67726	-3.48386
H	3.53694	3.15186	-0.32534
H	2.40600	2.14405	-4.31546
H	3.86522	3.44440	-2.77749
C	-1.11659	-0.99502	1.37663
C	-1.95976	-1.74639	0.54768
C	-1.33406	-1.18882	2.74131
C	-2.92631	-2.60771	1.03279
C	-2.29989	-2.04949	3.24325
C	-3.11084	-2.77215	2.39283
H	-1.88748	-1.68198	-0.52001
H	-0.76001	-0.67726	3.48386

H	-3.53694	-3.15186	0.32534
H	-2.40600	-2.14405	4.31546
H	-3.86522	-3.44440	2.77749
C	1.42000	-0.46948	1.37663
C	2.49229	-0.82400	0.54768
C	1.69658	-0.56092	2.74131
C	3.72150	-1.23040	1.03279
C	2.92486	-0.96702	3.24325
C	3.95618	-1.30799	2.39283
H	2.40037	-0.79361	-0.52001
H	0.96653	-0.31956	3.48386
H	4.49806	-1.48715	0.32534
H	3.05980	-1.01163	4.31546
H	4.91555	-1.62518	2.77749

Molecule 13

C	0.00000	0.00000	0.74077
C	-0.42330	1.11958	1.45444
C	0.42330	-1.11958	1.45444
C	-0.42352	1.12031	2.83963
C	0.42352	-1.12031	2.83963
C	0.00000	0.00000	3.53795
H	-0.77583	1.99072	0.91809
H	0.77583	-1.99072	0.91809
H	-0.76306	1.99652	3.37523
H	0.76306	-1.99652	3.37523
H	0.00000	0.00000	4.61929
C	0.00000	0.00000	-0.74077
C	-0.42330	-1.11958	-1.45444
C	0.42330	1.11958	-1.45444
C	-0.42352	-1.12031	-2.83963

C	0.42352	1.12031	-2.83963
C	0.00000	0.00000	-3.53795
H	-0.77583	-1.99072	-0.91809
H	0.77583	1.99072	-0.91809
H	-0.76306	-1.99652	-3.37523
H	0.76306	1.99652	-3.37523
H	0.00000	0.00000	-4.61929

Molecule 14

C	0.70144	1.65585	0.75774
C	0.70144	0.27110	0.12406
C	-0.70144	-0.27110	-0.12406
H	1.71528	2.02005	0.92440
H	0.18820	1.64454	1.72101
H	0.18820	2.37742	0.11949
H	1.24633	-0.42667	0.76619
H	1.24633	0.30090	-0.82374
C	-0.70144	-1.65585	-0.75774
H	-1.71528	-2.02005	-0.92440
H	-1.24633	-0.30090	0.82374
H	-1.24633	0.42667	-0.76619
H	-0.18820	-2.37742	-0.11949
H	-0.18820	-1.64454	-1.72101

Molecule 15

C	0.00000	0.00000	1.19377
C	0.00000	0.00000	-0.27821
C	-0.88540	1.15433	-0.76733
C	1.44238	0.18962	-0.76733
C	-0.55698	-1.34395	-0.76733

H	-0.89223	1.16323	-1.85770
H	-1.90979	1.03800	-0.41608
H	-0.50755	2.11356	-0.41608
H	1.45350	0.19108	-1.85770
H	1.85383	1.13493	-0.41608
H	2.08417	-0.61723	-0.41608
H	-0.56127	-1.35431	-1.85770
H	0.05596	-2.17293	-0.41608
H	-1.57663	-1.49633	-0.41608
N	0.00000	0.00000	2.34116

Molecule 16

C	-0.60510	-0.52264	0.16978
C	0.60510	0.52264	0.16978
C	0.09116	2.00655	0.02389
C	1.37471	0.37518	1.49689
C	1.62921	0.25416	-0.94124
H	2.31513	0.92272	1.45660
H	0.81397	0.74806	2.35169
H	1.62206	-0.66688	1.69559
H	2.38772	1.03910	-0.93679
H	2.14905	-0.68999	-0.78498
H	1.18441	0.23917	-1.93426
C	-0.09116	-2.00655	0.02389
C	-1.37471	-0.37518	1.49689
C	-1.62921	-0.25416	-0.94124
H	-2.31513	-0.92272	1.45660
H	-0.81397	-0.74806	2.35169
H	-1.62206	0.66688	1.69559
H	-2.38772	-1.03910	-0.93679
H	-2.14905	0.68999	-0.78498

H	-1.18441	-0.23917	-1.93426
C	0.97642	3.04287	0.72595
C	-0.09116	2.49284	-1.42088
H	-0.88530	2.06300	0.50748
H	0.58798	4.04340	0.53189
H	1.01431	2.91044	1.80483
H	2.00014	3.01571	0.34569
H	-0.62899	3.44193	-1.41780
H	0.87451	2.67237	-1.89590
H	-0.64606	1.80485	-2.05042
C	-0.97642	-3.04287	0.72595
C	0.09116	-2.49284	-1.42088
H	0.88530	-2.06300	0.50748
H	-0.58798	-4.04340	0.53189
H	-1.01431	-2.91044	1.80483
H	-2.00014	-3.01571	0.34569
H	0.62899	-3.44193	-1.41780
H	-0.87451	-2.67237	-1.89590
H	0.64606	-1.80485	-2.05042

Molecule 17

C	0.51961	0.63295	0.00000
C	-0.51961	-0.63295	0.00000
C	0.12788	-2.13458	0.00000
C	-1.44355	-0.55026	1.22946
C	-1.44355	-0.55026	-1.22946
H	-2.05742	-1.43961	1.32278
H	-0.90913	-0.42452	2.16806
H	-2.13630	0.27525	1.13441
H	-2.05742	-1.43961	-1.32278
H	-2.13630	0.27525	-1.13441

H	-0.90913	-0.42452	-2.16806
C	-0.12788	2.13458	0.00000
C	1.44355	0.55026	1.22946
C	1.44355	0.55026	-1.22946
H	2.05742	1.43961	1.32278
H	0.90913	0.42452	2.16806
H	2.13630	-0.27525	1.13441
H	2.05742	1.43961	-1.32278
H	2.13630	-0.27525	-1.13441
H	0.90913	0.42452	-2.16806
C	-0.99106	-3.21308	0.00000
C	0.97326	-2.47546	-1.24318
C	0.97326	-2.47546	1.24318
H	-0.51182	-4.19155	0.00000
H	-1.62593	-3.18262	0.88178
H	-1.62593	-3.18262	-0.88178
H	1.07027	-3.55917	-1.32268
H	0.51790	-2.12306	-2.16747
H	1.98322	-2.08223	-1.18952
H	1.07027	-3.55917	1.32268
H	1.98322	-2.08223	1.18952
H	0.51790	-2.12306	2.16747
C	0.99106	3.21308	0.00000
C	-0.97326	2.47546	-1.24318
C	-0.97326	2.47546	1.24318
H	0.51182	4.19155	0.00000
H	1.62593	3.18262	0.88178
H	1.62593	3.18262	-0.88178
H	-1.07027	3.55917	-1.32268
H	-0.51790	2.12306	-2.16747
H	-1.98322	2.08223	-1.18952
H	-1.07027	3.55917	1.32268

H	-1.98322	2.08223	1.18952
H	-0.51790	2.12306	2.16747

Molecule 18

C	-0.44540	-0.65793	-0.05783
C	0.44540	0.65793	-0.05783
C	-0.45280	1.90397	-0.26321
C	1.22833	0.77187	1.25746
C	1.46770	0.63108	-1.20535
H	1.94081	1.59536	1.20865
H	0.57555	0.95066	2.10999
H	1.80086	-0.13318	1.46065
H	1.96427	1.59785	-1.29096
H	2.24529	-0.11331	-1.04033
H	0.99737	0.42246	-2.16686
C	0.45280	-1.90397	-0.26321
C	-1.22833	-0.77187	1.25746
C	-1.46770	-0.63108	-1.20535
H	-1.94081	-1.59536	1.20865
H	-0.57555	-0.95066	2.10999
H	-1.80086	0.13318	1.46065
H	-1.96427	-1.59785	-1.29096
H	-2.24529	0.11331	-1.04033
H	-0.99737	-0.42246	-2.16686
C	0.18226	3.29212	-0.04158
H	-1.33557	1.83725	0.37742
H	-0.82562	1.86583	-1.28771
C	-0.32320	4.27888	-1.09237
C	-0.09199	3.84064	1.35801
H	1.26549	3.21743	-0.16386

H	-0.06094	3.95050	-2.09914
H	-1.41140	4.36777	-1.04357
H	0.09895	5.27329	-0.93883
H	0.38887	4.81001	1.49877
H	-1.16593	3.97827	1.50629
H	0.26818	3.17298	2.13871
C	-0.18226	-3.29212	-0.04158
H	1.33557	-1.83725	0.37742
H	0.82562	-1.86583	-1.28771
C	0.32320	-4.27888	-1.09237
C	0.09199	-3.84064	1.35801
H	-1.26549	-3.21743	-0.16386
H	0.06094	-3.95050	-2.09914
H	1.41140	-4.36777	-1.04357
H	-0.09895	-5.27329	-0.93883
H	-0.38887	-4.81001	1.49877
H	1.16593	-3.97827	1.50629
H	-0.26818	-3.17298	2.13871

Molecule 19

H	-2.23753	-3.38410	0.00419
H	3.54775	-0.30323	2.47670
C	-2.17136	-2.29405	0.00558
C	3.21920	-0.18344	1.44230
C	-1.16718	1.76431	1.23857
C	-1.28184	1.79425	-1.27796
C	-3.36068	1.87281	0.05842
H	-0.70964	-0.15351	2.11594
H	1.61669	-2.22315	-0.55354
H	-0.76692	-0.10211	-2.18851
H	1.19973	2.03910	-0.05962

H	-4.43365	0.00639	0.04225
H	4.90603	0.35951	-1.36241
C	-1.18298	0.23105	1.21207
C	1.85100	-1.22261	-0.90766
C	-1.22704	0.25489	-1.26152
C	1.64274	1.20364	-0.59522
C	-3.39482	0.34960	0.02725
C	3.81673	0.27171	-1.39164
C	-2.79681	-1.73386	1.27561
C	3.85200	-1.27053	0.57235
C	-2.85613	-1.73021	-1.23562
C	3.63964	1.19325	0.92481
C	-0.70694	-1.88984	-0.05351
C	1.69110	-0.28972	1.39741
C	-2.70918	-0.21349	-1.23257
C	3.16788	1.34466	-0.52146
C	-2.66274	-0.21824	1.25114
C	3.37804	-1.09414	-0.87221
C	-0.46207	-0.35813	-0.04271
C	1.13800	-0.14975	-0.03909
H	-2.29100	-2.14596	2.15321
H	3.56744	-2.25941	0.94166
H	-2.40139	-2.15490	-2.13463
H	3.20024	1.97704	1.54778
H	-3.85090	-2.01509	1.34681
H	4.94209	-1.20759	0.62378
H	-3.91586	-1.99869	-1.25158
H	4.72545	1.30579	0.98146
H	-0.29752	-2.31336	-0.96839
H	1.27872	0.49389	2.03465
H	-0.17102	-2.34909	0.78093
H	1.38112	-1.24508	1.82795

H	-3.20136	0.19824	-2.11831
H	3.43792	2.33521	-0.89349
H	-3.11030	0.20092	2.15744
H	3.80469	-1.88233	-1.49609
H	-1.66892	2.10614	2.14839
H	-0.15057	2.14923	1.28989
H	-0.31430	2.24680	-1.44510
H	-1.90661	2.10012	-2.12224
H	-3.90645	2.28285	-0.79537
H	-3.85277	2.23717	0.96436
C	-1.90358	2.32582	0.01579
H	-1.85182	3.41622	0.02574
H	1.49471	-1.15414	-1.94004
H	1.34945	1.29301	-1.64169
H	3.50855	0.39624	-2.43305

Molecule 20

C	-1.47675	0.63523	1.00654
C	-0.80847	0.15686	-0.32841
C	0.80852	-0.15741	-0.32799
C	2.48852	-1.63386	-1.62227
C	1.03218	-1.25226	-1.40228
C	1.47657	-0.63329	1.00796
C	1.68803	1.07790	-0.74292
C	2.94370	-1.07156	0.75012
C	3.15587	0.66073	-0.99910
C	3.04491	-2.16588	-0.30537
C	3.26167	-0.40459	-2.08061
C	3.77467	0.14157	0.30261
H	2.53552	-2.41214	-2.38694
H	3.34088	-1.44566	1.69836

H	3.69676	1.55730	-1.31760
H	0.49842	-2.15840	-1.12429
H	0.61088	-0.91289	-2.35156
C	1.54304	0.45152	2.09968
H	0.94859	-1.50676	1.39121
H	1.32673	1.48709	-1.68321
C	1.69665	2.18071	0.32315
H	4.08715	-2.47499	-0.42107
H	2.47890	-3.04795	0.00690
H	2.84745	-0.03404	-3.02236
H	4.31152	-0.64981	-2.26215
H	4.80119	-0.18399	0.10908
C	3.77515	1.24196	1.35532
C	-2.94386	1.07295	0.74763
C	-1.68786	-1.07927	-0.74113
C	-1.03201	1.24967	-1.40480
C	-3.15568	-0.66266	-0.99834
C	-2.48834	1.63078	-1.62575
C	-3.77470	-0.14106	0.30229
C	-3.04497	2.16527	-0.30995
C	-3.26136	0.40061	-2.08188
H	-3.34120	1.44883	1.69509
H	-3.69647	-1.55985	-1.31522
H	-2.53525	2.40760	-2.39190
C	-1.54337	-0.44751	2.10029
H	-0.94887	1.50944	1.38823
H	-1.32638	-1.49020	-1.68059
C	-1.69659	-2.18005	0.32703
H	-0.49833	2.15634	-1.12843
H	-0.61054	0.90851	-2.35336
H	-4.80121	0.18410	0.10799
C	-3.77532	-1.23946	1.35708

H	-2.47905	3.04796	0.00073
H	-4.08720	2.47411	-0.42640
H	-4.31119	0.64543	-2.26406
H	-2.84696	0.02830	-3.02286
C	2.33322	1.66415	1.61502
H	2.03006	0.02215	2.98018
H	0.56324	0.77799	2.42286
H	0.68785	2.53934	0.52823
H	2.26287	3.03709	-0.05375
H	4.36944	2.09112	1.00688
H	4.23271	0.88284	2.28103
H	2.30467	2.44576	2.37668
C	-2.33342	-1.66109	1.61780
H	-2.03055	-0.01649	2.97989
H	-0.56361	-0.77333	2.42426
H	-0.68778	-2.53818	0.53297
H	-2.26266	-3.03721	-0.04834
H	-4.36952	-2.08930	1.01016
H	-4.23302	-0.87860	2.28204
H	-2.30498	-2.44127	2.38094

Molecule 21

C	1.90249	-0.68451	-1.14636
C	1.34482	0.03515	0.10974
C	-0.30382	0.03013	0.26657
C	-2.20644	-1.27505	1.44313
C	-0.72190	-1.26315	1.05248
C	-1.20664	0.00346	-1.03545
C	-0.78092	1.30943	1.05165
C	-2.72082	-0.04810	-0.61578
C	-2.26642	1.19802	1.46047

C	-3.05251	-1.31393	0.16986
C	-2.53818	-0.05194	2.28095
C	-3.13015	1.17931	0.18823
H	-2.38871	-2.18127	2.02869
H	-3.29123	-0.05487	-1.55167
H	-2.51436	2.08211	2.05421
C	-0.45518	-2.53808	0.23941
H	-0.17272	-1.30716	1.98959
C	-1.02580	1.29139	-1.84849
C	-1.01839	-1.26449	-1.88434
H	-0.18550	1.39744	1.96568
C	-0.70227	2.63413	0.25979
H	-4.11379	-1.29342	0.43564
C	-2.75556	-2.55725	-0.65880
H	-1.92829	-0.04494	3.18879
H	-3.58525	-0.07649	2.59510
H	-4.18117	1.07959	0.47609
C	-2.95358	2.41580	-0.68231
C	3.43622	-0.72927	-1.17982
C	1.98958	-0.63004	1.35817
C	1.98551	1.44829	0.04989
C	3.52067	-0.65313	1.30771
C	3.51859	1.43935	-0.00383
C	3.96691	-1.43280	0.06964
C	3.99258	0.68951	-1.24463
C	4.07171	0.76769	1.24908
H	3.73793	-1.28601	-2.06954
H	3.88403	-1.15481	2.20709
H	3.85655	2.47705	-0.04675
H	1.54577	-1.70564	-1.21754
H	1.56038	-0.16436	-2.04187
H	1.66554	-0.09951	2.25811

H	1.66661	-1.66192	1.46403
H	1.63077	1.97973	-0.83130
H	1.70117	2.02169	0.92980
H	5.05754	-1.49578	0.03306
H	3.58563	-2.45668	0.11503
H	3.63683	1.19151	-2.14831
H	5.08470	0.67427	-1.28978
H	5.16460	0.75597	1.22749
H	3.77014	1.32477	2.14005
C	-1.47813	2.52561	-1.05117
H	-1.61845	1.21749	-2.76600
H	0.01383	1.40437	-2.16077
H	0.30170	2.97453	0.07091
H	-1.16299	3.41091	0.87691
H	-3.27040	3.31674	-0.15096
H	-3.57970	2.32637	-1.57436
H	-1.30927	3.42162	-1.65178
C	-1.28049	-2.52946	-1.05276
H	-0.75313	-3.40131	0.84154
H	0.60066	-2.67425	0.02922
H	-0.04736	-1.30305	-2.36034
H	-1.74652	-1.22853	-2.70120
H	-3.38877	-2.57132	-1.54973
H	-2.98088	-3.45992	-0.08457
H	-1.03736	-3.41137	-1.64868

Molecule 22

C	-1.89239	-1.02529	0.72477
C	-1.03120	0.18720	0.17801
C	0.61952	-0.02950	-0.12227
C	2.43728	0.57864	-1.86121

C	1.01618	0.86821	-1.34719
C	0.96157	-1.53375	-0.41254
C	1.69284	0.38023	0.99902
C	2.38482	-1.72896	-0.96542
C	3.14691	0.12328	0.44170
C	2.59713	-0.89101	-2.21632
C	3.44817	0.98011	-0.78607
C	3.39586	-1.34159	0.11631
H	2.59555	1.18994	-2.75479
H	2.49964	-2.78862	-1.21188
H	3.82644	0.41528	1.25000
H	0.35336	0.65950	-2.18240
C	0.95250	2.37039	-1.02196
C	0.86347	-2.44016	0.82882
H	0.29182	-1.89834	-1.18505
C	1.73923	1.88113	1.34783
C	1.55518	-0.50591	2.24417
H	3.59164	-1.07357	-2.63233
H	1.86979	-1.17330	-2.98308
C	3.35064	2.46209	-0.45124
H	4.45933	0.74803	-1.13401
H	4.41050	-1.45053	-0.27881
C	3.24710	-2.16726	1.38620
C	-3.34362	-0.57063	1.06423
C	-1.82051	0.58965	-1.12072
C	-1.20203	1.31679	1.22126
C	-3.25931	1.05514	-0.78704
C	-2.63555	1.73918	1.50881
C	-4.06070	-0.11842	-0.21607
C	-3.38598	0.55179	2.08772
C	-3.27113	2.21009	0.20567
H	-3.85736	-1.44663	1.47020

H	-3.71893	1.37852	-1.72609
H	-2.61369	2.55940	2.22978
C	-2.08418	-2.20482	-0.24856
H	-1.46324	-1.37188	1.66427
H	-1.34364	1.43583	-1.60581
C	-1.92667	-0.56331	-2.13023
H	-0.74571	1.00483	2.15918
H	-0.67997	2.20341	0.89343
H	-5.06704	0.22443	0.04261
C	-4.14968	-1.24709	-1.23421
H	-2.91601	0.23453	3.02263
H	-4.42312	0.81105	2.31690
H	-4.29775	2.54651	0.37279
H	-2.71308	3.06178	-0.19362
C	1.82956	-1.97858	1.91359
H	1.11750	-3.46003	0.52466
H	-0.13305	-2.49011	1.23913
H	0.55922	-0.41189	2.67990
H	2.26297	-0.16124	3.00480
H	3.98628	-1.84286	2.12375
H	3.43060	-3.22594	1.18508
H	1.68362	-2.58091	2.81252
C	-2.73528	-1.72427	-1.54631
H	-2.74020	-2.93537	0.23338
H	-1.16870	-2.73445	-0.47005
H	-0.94182	-0.90988	-2.43989
H	-2.42080	-0.19414	-3.03352
H	-4.64715	-0.89435	-2.14182
H	-4.74622	-2.07168	-0.83531
H	-2.76824	-2.54589	-2.26435
C	1.94812	2.73337	0.08592
H	-0.05190	2.69136	-0.76576

H	1.21840	2.92632	-1.92583
H	2.59865	2.03220	2.00863
H	0.88297	2.21390	1.91764
H	3.54482	3.06707	-1.34097
H	4.10441	2.72726	0.29453
H	1.84304	3.79006	0.33950

Molecule 23

C	1.90126	0.28489	1.02672
C	0.89897	-0.08929	-0.16136
C	-0.89900	-0.08733	0.16043
C	-2.77583	0.74649	1.76756
C	-1.30604	0.91550	1.30716
C	-1.39745	-1.54036	0.55751
C	-1.90152	0.28774	-1.02731
C	-2.86291	-1.59668	1.04181
C	-3.40281	0.17419	-0.53321
C	-3.07632	-0.66720	2.21836
C	-3.71135	1.13129	0.61198
C	-3.78974	-1.23257	-0.12057
H	-2.92802	1.43376	2.60396
H	-3.05168	-2.62652	1.35675
H	-4.00845	0.45815	-1.40099
H	-0.67868	0.73149	2.17908
C	-1.23621	2.40343	0.93031
C	-1.37240	-2.59431	-0.57610
H	-0.81659	-1.86826	1.41245
C	-1.82186	1.74990	-1.48157
C	-1.76909	-0.70690	-2.18834
H	-4.10260	-0.74047	2.58859
H	-2.41373	-0.94989	3.04146

C	-3.53647	2.56591	0.14003
H	-4.74715	0.97410	0.92749
H	-4.82704	-1.22953	0.22752
C	-3.65556	-2.14878	-1.32702
C	3.40279	0.16916	0.53376
C	1.30827	0.91233	-1.30836
C	1.39550	-1.54350	-0.55720
C	2.77815	0.74096	-1.76763
C	2.86120	-1.60193	-1.04068
C	3.71329	1.12530	-0.61157
C	3.78806	-1.23828	0.12191
C	3.07673	-0.67343	-2.21757
H	4.00823	0.45269	1.40181
H	2.93188	1.42748	-2.60435
H	3.04879	-2.63220	-1.35491
C	1.82226	1.74730	1.48023
C	1.76676	-0.70903	2.18814
H	0.68146	0.72861	-2.18072
C	1.24028	2.40062	-0.93259
C	1.36932	-2.59696	0.57687
H	0.81439	-1.87143	-1.41202
H	4.74917	0.96677	-0.92618
C	3.53975	2.56036	-0.14035
C	3.65230	-2.15356	1.32885
H	4.82552	-1.23671	-0.22573
H	4.10313	-0.74845	-2.58712
H	2.41423	-0.95554	-3.04094
C	-2.19801	-2.12213	-1.76853
H	-1.82145	-3.50837	-0.17760
H	-0.39377	-2.88638	-0.90626
H	-0.74462	-0.72584	-2.56358
H	-2.39844	-0.37013	-3.01828

H	-4.32087	-1.80168	-2.12249
H	-3.94802	-3.17239	-1.08011
H	-2.04870	-2.80374	-2.60832
C	2.09101	2.71160	0.30543
H	2.60314	1.90397	2.23084
H	0.89311	1.96534	1.98890
H	0.23929	2.76228	-0.82556
H	1.65011	2.96646	-1.77396
H	3.76599	3.26658	-0.94292
H	4.23198	2.76373	0.68093
H	1.90002	3.73686	0.63081
C	-2.08797	2.71498	-0.30683
H	-0.23470	2.76353	0.82179
H	-1.64416	2.97022	1.77198
H	-2.60376	1.90680	-2.23109
H	-0.89342	1.96694	-1.99185
H	-3.76122	3.27291	0.94236
H	-4.22909	2.76976	-0.68079
H	-1.89577	3.73982	-0.63289
C	2.19460	-2.12495	1.76952
H	0.74193	-0.72668	2.56246
H	2.39569	-0.37238	3.01846
H	1.81810	-3.51138	0.17887
H	0.39052	-2.88844	0.90669
H	4.31758	-1.80674	2.12446
H	3.94369	-3.17765	1.08269
H	2.04408	-2.80598	2.60957

Molecule 24

C	-1.88216	-1.36819	0.24108
C	-1.39416	0.13465	0.35043

C	0.29971	0.47500	0.22809
C	2.60912	-0.36957	1.08774
C	1.11481	-0.72677	0.82664
C	0.59400	1.77604	1.07607
C	1.01016	0.78967	-1.15775
C	2.08730	2.05199	1.28800
C	2.53126	1.10957	-0.90968
C	2.73294	0.86201	1.96966
C	3.26166	-0.06289	-0.27436
C	2.73715	2.35318	-0.05725
C	3.33662	-1.55133	1.73479
H	2.16490	2.92990	1.93702
H	2.95890	1.28937	-1.90258
H	0.67395	-1.00501	1.79032
C	1.12489	-1.97274	-0.08532
C	-0.01479	3.03380	0.44648
H	0.21677	1.65104	2.08168
C	1.05159	-0.42459	-2.09213
C	0.44809	2.05791	-1.82300
H	3.79012	1.06122	2.17096
H	2.25199	0.67628	2.93565
C	3.23609	-1.28954	-1.18042
H	4.30432	0.22740	-0.09765
H	3.81048	2.51925	0.07565
C	2.10410	3.56963	-0.71915
C	-3.34702	-1.52981	0.74709
C	-2.39520	0.86488	-0.62080
C	-1.73498	0.57972	1.80393
C	-3.85855	0.73342	-0.10061
C	-3.17374	0.39304	2.25202
C	-4.30126	-0.73574	-0.15643
C	-3.53103	-1.07988	2.18446

C	-4.04423	1.23476	1.32534
H	-3.58674	-2.59413	0.66858
H	-4.48777	1.32123	-0.77548
H	-3.26201	0.75785	3.27776
C	-1.91097	-1.95422	-1.18413
H	-1.27216	-1.99168	0.89455
H	-2.17337	1.92721	-0.66653
C	-2.41045	0.31229	-2.05600
H	-1.07427	0.05733	2.49971
H	-1.54851	1.64033	1.91461
H	-5.31610	-0.81428	0.24469
C	-4.27210	-1.26732	-1.57998
H	-2.88026	-1.65064	2.85262
H	-4.56150	-1.25625	2.50403
H	-5.09879	1.16593	1.60464
H	-3.75673	2.28671	1.40558
C	0.61851	3.28783	-0.92321
H	0.18943	3.88579	1.10144
H	-1.09562	2.96847	0.36657
H	-0.59637	1.95260	-2.08638
H	0.98617	2.22137	-2.76241
H	2.58973	3.76639	-1.67867
H	2.24149	4.45771	-0.09639
H	0.13903	4.14650	-1.39744
C	-2.84333	-1.15091	-2.08825
H	-2.25926	-2.98909	-1.11971
H	-0.93323	-1.99768	-1.64278
H	-1.44449	0.40821	-2.53464
H	-3.10604	0.91497	-2.64736
H	-4.95344	-0.69339	-2.21382
H	-4.60380	-2.30883	-1.60208
H	-2.77357	-1.52964	-3.10999

C	1.76283	-1.61710	-1.43546
H	0.12261	-2.30950	-0.26137
C	1.85400	-3.14270	0.57585
H	1.59509	-0.14217	-2.99959
H	0.06004	-0.71154	-2.41984
C	3.96988	-2.45217	-0.51377
H	3.72444	-1.04165	-2.12763
H	1.69082	-2.48452	-2.09926
C	3.30985	-2.77858	0.82814
H	2.87341	-1.78490	2.69866
H	4.36975	-1.25486	1.94125
H	1.78679	-4.02178	-0.07124
H	1.35669	-3.39830	1.51608
H	5.01959	-2.18539	-0.36342
H	3.95068	-3.33215	-1.16243
H	3.83336	-3.60779	1.30786

Molecule 25

C	0.00000	0.82114	1.37722
C	0.00000	0.82114	-1.37722
C	0.00000	-0.82114	1.37722
C	0.00000	-0.82114	-1.37722
C	0.00000	0.76657	2.91810
C	0.00000	0.76657	-2.91810
C	0.00000	-0.76657	2.91810
C	0.00000	-0.76657	-2.91810
C	1.22093	1.40821	-0.70069
C	1.22093	1.40821	0.70069
C	2.31770	1.91252	-1.37748
C	2.31770	1.91252	1.37748
C	3.41184	2.41909	-0.68972

C	3.41184	2.41909	0.68972
H	2.34217	1.91910	-2.45660
H	2.34217	1.91910	2.45660
H	4.25827	2.80907	-1.23836
H	4.25827	2.80907	1.23836
C	-1.22093	1.40821	0.70069
C	-1.22093	1.40821	-0.70069
C	-2.31770	1.91252	1.37748
C	-2.31770	1.91252	-1.37748
C	-3.41184	2.41909	0.68972
C	-3.41184	2.41909	-0.68972
H	-2.34217	1.91910	2.45660
H	-2.34217	1.91910	-2.45660
H	-4.25827	2.80907	1.23836
H	-4.25827	2.80907	-1.23836
C	1.22093	-1.40821	0.70069
C	1.22093	-1.40821	-0.70069
C	2.31770	-1.91252	1.37748
C	2.31770	-1.91252	-1.37748
C	3.41184	-2.41909	0.68972
C	3.41184	-2.41909	-0.68972
H	2.34217	-1.91910	2.45660
H	2.34217	-1.91910	-2.45660
H	4.25827	-2.80907	1.23836
H	4.25827	-2.80907	-1.23836
C	-1.22093	-1.40821	-0.70069
C	-1.22093	-1.40821	0.70069
C	-2.31770	-1.91252	-1.37748
C	-2.31770	-1.91252	1.37748
C	-3.41184	-2.41909	-0.68972
C	-3.41184	-2.41909	0.68972
H	-2.34217	-1.91910	-2.45660

H	-2.34217	-1.91910	2.45660
H	-4.25827	-2.80907	-1.23836
H	-4.25827	-2.80907	1.23836
H	0.87130	1.20898	3.38822
H	-0.87130	1.20898	3.38822
H	-0.87130	1.20898	-3.38822
H	0.87130	1.20898	-3.38822
H	-0.87130	-1.20898	3.38822
H	0.87130	-1.20898	3.38822
H	0.87130	-1.20898	-3.38822
H	-0.87130	-1.20898	-3.38822

Molecule 26

C	-3.26758	-0.11859	-0.00084
C	-1.89505	-0.06933	-0.00076
C	-4.00258	-0.22921	1.17860
C	-1.16634	-0.09709	1.17951
C	-3.29690	-0.29354	2.35573
C	-1.86920	-0.23031	2.35282
C	-1.16998	0.01105	-1.18088
C	-4.00849	-0.06073	-1.18033
C	-1.88052	0.09344	-2.35429
C	-3.30910	0.05407	-2.35735
C	-5.46795	-0.25556	0.78395
C	-5.47202	-0.13922	-0.78576
C	0.32538	-0.01923	0.85796
C	0.32343	0.04157	-0.85883
H	-3.80187	-0.39258	3.30811
H	-1.34890	-0.29271	3.30020
H	-1.36601	0.19334	-3.30162
H	-3.81978	0.11688	-3.30977

H	-6.01739	0.56868	1.23881
H	-5.95066	-1.17719	1.10911
H	-5.96117	-1.00058	-1.24066
H	-6.01935	0.74557	-1.11091
C	0.95396	-1.30693	1.42022
C	0.35664	-2.53135	1.10875
C	2.05240	-1.32779	2.26970
C	0.86631	-3.72473	1.57818
C	2.56440	-2.52646	2.75265
C	1.98483	-3.73036	2.40095
H	-0.51954	-2.55136	0.47639
H	2.52257	-0.40755	2.57776
H	0.38794	-4.65456	1.30211
H	3.42173	-2.50773	3.41218
H	2.38864	-4.66306	2.77065
C	0.96393	1.24326	1.44090
C	2.30156	1.55092	1.18626
C	0.23862	2.14924	2.20342
C	2.88589	2.70711	1.66507
C	0.81869	3.31343	2.69080
C	2.14366	3.60101	2.42352
H	2.89980	0.88718	0.58272
H	-0.80413	1.96396	2.41086
H	3.92192	2.91568	1.43560
H	0.22079	3.99890	3.27635
H	2.59462	4.51096	2.79514
C	0.85755	1.37182	-1.41976
C	0.17182	2.54938	-1.10960
C	1.95441	1.47314	-2.26551
C	0.59512	3.77703	-1.57648
C	2.37961	2.70623	-2.74596
C	1.71306	3.86458	-2.39536

H	-0.70612	2.50527	-0.48094
H	2.49133	0.58982	-2.57247
H	0.04949	4.66943	-1.30161
H	3.23829	2.75037	-3.40253
H	2.04948	4.82447	-2.76295
C	1.05277	-1.17069	-1.44185
C	2.40839	-1.38128	-1.18372
C	0.39717	-2.12522	-2.20830
C	3.07634	-2.49111	-1.66286
C	1.06149	-3.24334	-2.69605
C	2.40303	-3.43480	-2.42530
H	2.95510	-0.67743	-0.57672
H	-0.65561	-2.01566	-2.41882
H	4.12404	-2.62465	-1.43046
H	0.51647	-3.96910	-3.28467
H	2.91979	-4.30894	-2.79720

Molecule 27

C	0.81900	0.10540	0.00000
C	-0.81900	-0.10540	0.00000
O	1.50793	-1.05622	0.00000
O	-1.50793	1.05622	0.00000
N	0.53644	2.15368	-1.37508
N	1.69443	0.59971	-2.37597
C	1.06854	0.95419	-1.23578
C	0.82383	2.60539	-2.64002
C	1.54141	1.63868	-3.27753
C	0.37256	3.93901	-3.11852
C	2.11790	1.58917	-4.64645
C	2.37017	-0.64503	-2.70396
N	1.69443	0.59971	2.37597

N	0.53644	2.15368	1.37508
C	1.06854	0.95419	1.23578
C	1.54141	1.63868	3.27753
C	0.82383	2.60539	2.64002
C	2.11790	1.58917	4.64645
C	0.37256	3.93901	3.11852
C	2.37017	-0.64503	2.70396
N	-0.53644	-2.15368	-1.37508
N	-1.69443	-0.59971	-2.37597
C	-1.06854	-0.95419	-1.23578
C	-0.82383	-2.60539	-2.64002
C	-1.54141	-1.63868	-3.27753
C	-0.37256	-3.93901	-3.11852
C	-2.11790	-1.58917	-4.64645
C	-2.37017	0.64503	-2.70396
N	-1.69443	-0.59971	2.37597
N	-0.53644	-2.15368	1.37508
C	-1.06854	-0.95419	1.23578
C	-1.54141	-1.63868	3.27753
C	-0.82383	-2.60539	2.64002
C	-2.11790	-1.58917	4.64645
C	-0.37256	-3.93901	3.11852
C	-2.37017	0.64503	2.70396
Fe	0.65942	-2.79365	0.00000
Cl	1.11265	-4.95995	0.00000
Fe	-0.65942	2.79365	0.00000
Cl	-1.11265	4.95995	0.00000
H	0.71955	4.12762	-4.13256
H	-0.71550	4.01461	-3.10548
H	0.74401	4.72867	-2.46653
H	1.77178	0.71432	-5.19967
H	1.82141	2.47469	-5.20316

H	3.20914	1.55899	-4.62901
H	2.61944	-1.15772	-1.78522
H	1.71389	-1.27664	-3.30370
H	3.27115	-0.42178	-3.27067
H	1.77178	0.71432	5.19967
H	3.20914	1.55899	4.62901
H	1.82141	2.47469	5.20316
H	0.71955	4.12762	4.13256
H	0.74401	4.72867	2.46653
H	-0.71550	4.01461	3.10548
H	2.61944	-1.15772	1.78522
H	3.27115	-0.42178	3.27067
H	1.71389	-1.27664	3.30370
H	-0.71955	-4.12762	-4.13256
H	0.71550	-4.01461	-3.10548
H	-0.74401	-4.72867	-2.46653
H	-1.77178	-0.71432	-5.19967
H	-1.82141	-2.47469	-5.20316
H	-3.20914	-1.55899	-4.62901
H	-2.61944	1.15772	-1.78522
H	-1.71389	1.27664	-3.30370
H	-3.27115	0.42178	-3.27067
H	-1.77178	-0.71432	5.19967
H	-3.20914	-1.55899	4.62901
H	-1.82141	-2.47469	5.20316
H	-0.71955	-4.12762	4.13256
H	-0.74401	-4.72867	2.46653
H	0.71550	-4.01461	3.10548
H	-2.61944	1.15772	1.78522
H	-3.27115	0.42178	3.27067
H	-1.71389	1.27664	3.30370

Molecule 28

C	0.00000	0.00000	1.76608
C	0.00000	0.00000	0.25452
H	0.00626	-1.02610	2.12701
Cl	-0.01024	1.67644	-0.36284
H	-0.89176	0.50762	2.12701
H	0.88549	0.51848	2.12701
Cl	-1.44672	-0.84709	-0.36284
Cl	1.45696	-0.82936	-0.36284

Molecule 29

C	0.00000	0.00000	0.79129
C	0.00000	0.00000	-0.79129
Cl	-0.73621	1.49659	1.39544
Cl	0.73621	-1.49659	-1.39544
Cl	1.66419	-0.11072	1.39544
Cl	-0.92798	-1.38587	1.39544
Cl	0.92798	1.38587	-1.39544
Cl	-1.66419	0.11072	-1.39544

Molecule 30a

C	0.00000	0.00000	0.96757
C	0.00000	0.00000	-0.96757
H	0.50580	0.94747	1.11920
H	-0.50580	-0.94747	-1.11920
H	0.56763	-0.91178	1.11920
H	-1.07344	-0.03569	1.11920
H	1.07344	0.03569	-1.11920
H	-0.56763	0.91178	-1.11920

Molecule 30b

C	0.00000	0.79554	0.00000
C	0.00000	-0.79554	0.00000
H	1.12765	0.65953	0.02337
H	-1.12765	-0.65953	-0.02337
H	-0.36356	1.25612	0.91085
H	-0.32551	1.25612	-0.92513
H	0.32551	-1.25612	0.92513
H	0.36356	-1.25612	-0.91085

Molecule 31

C	0.00000	0.96499	-0.15369
C	0.00000	-0.96499	-0.15369
B	-1.35450	0.00000	0.26231
B	-0.88232	1.45269	1.18859
B	0.88232	1.45269	1.18859
B	1.35450	0.00000	0.26231
B	-0.88232	-1.45269	1.18859
B	-1.43195	0.00000	2.03010
B	0.00000	0.88016	2.61431
B	1.43195	0.00000	2.03010
B	0.88232	-1.45269	1.18859
B	0.00000	-0.88016	2.61431
H	-2.29328	0.00000	-0.44820
H	-1.52783	2.43577	1.06266
H	1.52783	2.43577	1.06266
H	2.29328	0.00000	-0.44820
H	-1.52783	-2.43577	1.06266
H	-2.47533	0.00000	2.59033

H	0.00000	1.51879	3.61201
H	2.47533	0.00000	2.59033
H	1.52783	-2.43577	1.06266
H	0.00000	-1.51879	3.61201
N	0.00000	1.57347	-1.39849
N	0.00000	-1.57347	-1.39849
C	-1.20192	2.27055	-1.82473
H	-1.28026	3.26184	-1.36328
H	-1.16572	2.39377	-2.90621
H	-2.09052	1.70207	-1.57577
C	1.20192	2.27055	-1.82473
H	1.16572	2.39377	-2.90621
H	1.28026	3.26184	-1.36328
H	2.09052	1.70207	-1.57577
C	1.20192	-2.27055	-1.82473
H	1.28026	-3.26184	-1.36328
H	1.16572	-2.39377	-2.90621
H	2.09052	-1.70207	-1.57577
C	-1.20192	-2.27055	-1.82473
H	-1.16572	-2.39377	-2.90621
H	-1.28026	-3.26184	-1.36328
H	-2.09052	-1.70207	-1.57577

Molecule 32

C	1.28406	0.15062	0.00000
C	0.00000	0.47238	0.00000
H	1.60713	-0.88396	0.00000
H	-0.27557	1.52264	0.00000
H	2.05663	0.90758	0.00000
C	-1.13148	-0.50430	0.00000
H	-0.77011	-1.53228	0.00000

H	-1.76677	-0.36310	0.87673
H	-1.76677	-0.36310	-0.87673

Molecule 33

C	0.00000	0.00000	0.66090
C	0.00000	0.00000	-0.66090
H	0.00000	0.92183	1.22824
H	0.00000	-0.92183	-1.22824
H	0.00000	-0.92183	1.22824
H	0.00000	0.92183	-1.22824

Molecule 34

C	0.60379	1.60245	0.66807
C	0.60379	0.37596	0.15674
H	-0.32217	2.12157	0.88450
H	1.54777	-0.11877	-0.04952
H	1.52463	2.12494	0.88590
C	-0.60379	-0.37596	-0.15674
C	-0.60379	-1.60245	-0.66807
H	0.32217	-2.12157	-0.88450
H	-1.54777	0.11877	0.04952
H	-1.52463	-2.12494	-0.88590

Molecule 35

C	-1.71567	-0.24833	-0.29111
C	-0.53741	0.52308	0.30313
C	0.71724	-0.29274	0.33817
H	-2.62461	0.35291	-0.27637
H	-1.90923	-1.16188	0.27327

H	-1.51083	-0.53228	-1.32357
H	-0.79174	0.82937	1.32181
H	-0.36289	1.43751	-0.26700
C	1.84812	0.01616	-0.27677
H	2.71849	-0.62335	-0.21784
H	0.66468	-1.21580	0.90992
H	1.94247	0.92445	-0.86074

Molecule 36

C	0.02891	1.16959	1.25903
C	-0.31153	0.36972	0.00000
C	0.41202	-0.94478	-0.00000
C	-0.16064	-2.13793	-0.00000
C	0.02891	1.16959	-1.25903
H	-0.24310	0.62031	2.16011
H	1.10001	1.37988	1.30304
H	-0.49871	2.12439	1.26527
H	1.10001	1.37989	-1.30303
H	-0.24309	0.62032	-2.16011
H	-0.49871	2.12439	-1.26526
H	0.42414	-3.04790	-0.00000
H	1.49818	-0.87830	0.00000
H	-1.23924	-2.24543	-0.00001
H	-1.38553	0.16527	-0.00000

Molecule 37

C	-0.03153	0.34994	0.00000
C	-0.56566	-1.06160	0.00000
C	0.12810	-2.18904	0.00000
C	-0.56566	1.06355	-1.25047

C	-0.56566	1.06355	1.25047
C	1.49447	0.39823	0.00000
H	-0.24621	2.10733	-1.25958
H	-0.19779	0.58415	-2.15835
H	-1.65663	1.04522	-1.27638
H	-0.24621	2.10733	1.25958
H	-1.65663	1.04522	1.27638
H	-0.19779	0.58415	2.15835
H	1.83508	1.43463	0.00000
H	1.90744	-0.08975	0.88399
H	1.90744	-0.08975	-0.88399
H	-0.37155	-3.14835	0.00000
H	-1.65172	-1.12411	0.00000
H	1.21022	-2.20392	0.00000

Molecule 38

C	0.00000	0.00000	0.59694
C	0.00000	0.00000	-0.59694
H	0.00000	0.00000	1.65944
H	0.00000	0.00000	-1.65944

Molecule 39

C	0.00000	0.00000	1.41525
C	0.00000	0.00000	0.21893
H	0.00000	0.00000	2.47703
C	0.00000	0.00000	-1.23632
H	0.57395	0.84245	-1.62140
H	0.44261	-0.91828	-1.62140
H	-1.01656	0.07583	-1.62140

Molecule 40

C	0.00000	0.00000	-1.17355
C	0.00000	0.00000	0.28095
H	0.17758	1.00737	-1.54489
H	0.78362	-0.65748	-1.54489
H	-0.96120	-0.34990	-1.54489
N	0.00000	0.00000	1.42718

Molecule 41

C	0.37680	-1.64611	0.00000
C	-0.49231	-0.64107	0.00000
H	1.44364	-1.47013	0.00000
H	-1.55679	-0.84425	0.00000
H	0.03479	-2.67131	0.00000
C	-0.11702	0.73379	0.00000
C	0.17285	1.89675	0.00000
H	0.43647	2.92553	0.00000

Molecule 42

C	1.17853	-1.13099	0.00000
C	-0.09741	-0.76885	0.00000
H	1.97517	-0.40045	0.00000
H	-0.89449	-1.50016	0.00000
H	1.45159	-2.17636	0.00000
C	-0.50687	0.60097	0.00000
N	-0.85397	1.69574	0.00000

Molecule 43

C	0.00000	0.00000	1.88501

C	0.00000	0.00000	0.68601
H	0.00000	0.00000	2.94728
C	0.00000	0.00000	-0.68601
C	0.00000	0.00000	-1.88501
H	0.00000	0.00000	-2.94728

Molecule 44

C	0.00000	0.00000	-1.83029
C	0.00000	0.00000	-0.63431
H	0.00000	0.00000	-2.89363
C	0.00000	0.00000	0.74097
N	0.00000	0.00000	1.89077

Molecule 45

C	-0.00426	-0.90722	0.00000
C	-0.00741	-0.19332	1.19420
C	-0.00741	-0.19332	-1.19420
C	-0.00741	1.19293	1.19717
C	-0.00741	1.19293	-1.19717
C	-0.00619	1.89210	0.00000
C	0.02800	-2.41053	0.00000
H	-0.01198	-0.73075	2.13455
H	-0.01198	-0.73075	-2.13455
H	-0.01136	1.72824	2.13722
H	-0.01136	1.72824	-2.13722
H	-0.00908	2.97345	0.00000
H	1.05719	-2.77508	0.00000
H	-0.46441	-2.81731	0.88256
H	-0.46441	-2.81731	-0.88256

Molecule 46

C	-0.43305	-0.00005	-0.33327
C	0.26505	-1.19449	-0.18767
C	0.26498	1.19444	-0.18774
C	1.62209	-1.19768	0.09528
C	1.62201	1.19774	0.09521
C	2.30572	0.00005	0.23841
C	-1.91655	-0.00010	-0.59133
H	-0.26169	-2.13441	-0.30114
H	-0.26183	2.13432	-0.30126
H	2.14760	-2.13733	0.20097
H	2.14746	2.13743	0.20085
H	3.36492	0.00009	0.45641
H	-2.18255	0.87570	-1.18588
H	-2.18253	-0.87607	-1.18564
C	-2.72741	0.00007	0.70541
H	-2.49535	0.88057	1.30546
H	-3.79781	0.00004	0.49871
H	-2.49533	-0.88025	1.30570

Molecule 47

C	-0.67552	1.54813	0.00000
H	-1.76818	1.53806	0.00000
C	-0.21823	2.28396	1.26094
C	-0.21823	2.28396	-1.26094
H	-0.60737	3.30301	1.27281
H	-0.56263	1.77380	2.16036
H	0.87070	2.34291	1.30576
H	-0.60737	3.30301	-1.27281
H	0.87070	2.34291	-1.30576

H	-0.56263	1.77380	-2.16036
C	-0.21823	0.10491	0.00000
C	-1.14213	-0.93330	0.00000
C	1.13606	-0.22136	0.00000
C	-0.73206	-2.25900	0.00000
C	1.55183	-1.54224	0.00000
C	0.61781	-2.56838	0.00000
H	-2.20024	-0.70137	0.00000
H	1.87882	0.56666	0.00000
H	-1.47013	-3.04991	0.00000
H	2.60884	-1.77272	0.00000
H	0.94163	-3.60017	0.00000

Molecule 48

C	0.51664	-1.35804	0.00000
C	0.00234	-2.08175	1.25397
C	0.00234	-2.08175	-1.25397
C	2.04497	-1.43125	0.00000
H	-1.08662	-2.10882	1.28617
H	0.36297	-3.11192	1.26906
H	0.35251	-1.58332	2.15871
H	0.35251	-1.58332	-2.15871
H	0.36297	-3.11192	-1.26906
H	-1.08662	-2.10882	-1.28617
H	2.47249	-0.95802	0.88491
H	2.35856	-2.47582	0.00000
H	2.47249	-0.95802	-0.88491
C	0.00234	0.08296	0.00000
C	0.84763	1.18737	0.00000
C	-1.37230	0.32659	0.00000
C	0.34406	2.48204	0.00000

C	-1.88007	1.61332	0.00000
C	-1.02108	2.70284	0.00000
H	1.91933	1.05387	0.00000
H	-2.06573	-0.50417	0.00000
H	1.02906	3.31946	0.00000
H	-2.95101	1.76658	0.00000
H	-1.41410	3.71026	0.00000

Molecule 49

C	1.57678	0.62265	-0.00001
C	2.58333	-0.21342	-0.74704
C	2.58331	-0.21336	0.74709
H	1.76792	1.68741	-0.00005
H	3.38142	0.30175	-1.26145
H	2.21207	-1.09153	-1.25674
H	3.38139	0.30184	1.26147
H	2.21205	-1.09144	1.25685
C	0.13231	0.27590	-0.00001
C	-0.31854	-1.04306	0.00003
C	-0.81804	1.29270	-0.00005
C	-1.67251	-1.33274	0.00003
C	-2.17449	1.00616	-0.00004
C	-2.60882	-0.30928	-0.00000
H	0.39233	-1.86005	0.00005
H	-0.48953	2.32456	-0.00007
H	-1.99854	-2.36426	0.00006
H	-2.89302	1.81484	-0.00007
H	-3.66595	-0.53641	-0.00000

Molecule 50

C	0.50925	-0.21984	-0.04251
C	-0.40179	-1.27354	0.00193
C	0.00919	1.08301	-0.05974
C	-1.76709	-1.03956	0.04330
C	-1.35271	1.31942	-0.01925
C	-2.24789	0.25932	0.03488
C	1.94858	-0.52290	-0.07061
H	-0.03211	-2.29137	0.00859
H	0.68947	1.92183	-0.11703
H	-2.45491	-1.87334	0.08063
H	-1.72043	2.33650	-0.03577
H	-3.31237	0.44721	0.06438
C	2.94854	0.33415	0.09459
H	2.19025	-1.56854	-0.23241
H	2.78832	1.38819	0.28076
H	3.97533	-0.00089	0.05533

Molecule 51

C	0.00000	0.00000	0.58538
C	0.00000	1.20427	-0.11928
C	0.00000	-1.20427	-0.11928
C	0.00000	1.20047	-1.50316
C	0.00000	-1.20047	-1.50316
C	0.00000	0.00000	-2.19803
C	0.00000	0.00000	2.01571
H	0.00000	2.13685	0.42706
H	0.00000	-2.13685	0.42706
H	0.00000	2.13829	-2.04135
H	0.00000	-2.13829	-2.04135
H	0.00000	0.00000	-3.27934
C	0.00000	0.00000	3.21383

H	0.00000	0.00000	4.27586

Molecule 52

C	0.00000	0.00000	0.60245
C	0.00000	1.20857	-0.09040
C	0.00000	-1.20857	-0.09040
C	0.00000	1.20268	-1.47387
C	0.00000	-1.20268	-1.47387
C	0.00000	0.00000	-2.16513
C	0.00000	0.00000	2.03589
H	0.00000	2.13957	0.45798
H	0.00000	-2.13957	0.45798
H	0.00000	2.13900	-2.01371
H	0.00000	-2.13900	-2.01371
H	0.00000	0.00000	-3.24634
N	0.00000	0.00000	3.18426

Molecule 53

C	0.00000	0.00000	0.74077
C	-0.42330	1.11958	1.45444
C	0.42330	-1.11958	1.45444
C	-0.42352	1.12031	2.83963
C	0.42352	-1.12031	2.83963
C	0.00000	0.00000	3.53795
H	-0.77583	1.99072	0.91809
H	0.77583	-1.99072	0.91809
H	-0.76306	1.99652	3.37523
H	0.76306	-1.99652	3.37523
H	0.00000	0.00000	4.61929
C	0.00000	0.00000	-0.74077

C	-0.42330	-1.11958	-1.45444
C	0.42330	1.11958	-1.45444
C	-0.42352	-1.12031	-2.83963
C	0.42352	1.12031	-2.83963
C	0.00000	0.00000	-3.53795
H	-0.77583	-1.99072	-0.91809
H	0.77583	1.99072	-0.91809
H	-0.76306	-1.99652	-3.37523
H	0.76306	1.99652	-3.37523
H	0.00000	0.00000	-4.61929

REFERENCES

[1]C. Costain, "Determination of Molecular Structures from Ground State Rotational Constants," J. Chem. Phys. **29**, 864–874 (1958).

[2]R. Hilderbrandt and J. Wieser, "The Zero Point Average Structure of Isobutane as Determined by Electron Diffraction and Microwave Spectroscopy," J. Mol. Struct. **15**, 27–36 (1973).

[3]D. Lide, "Structure of the Isobutane Molecule; Change of Dipole Moment on Isotopic Substitution," J. Chem. Phys. **33**, 1519–1522 (1960).

[4]D. VanHemelrijk, L. Van den Enden, H. Geise, H. Sellers, and Schäfer, "Structure Determination of 1-Butene by Gas Electron Diffraction, Microwave Spectroscopy, Molecular Mechanics, and Molecular Orbital Constrained Electron Diffraction," J. Amer. Chem. Soc. **102**, 2189–2195 (1980).

[5]C. Bock and Y. Panchenko, "An Ab Initio Structural Investigation of 1,3-Butadiene, Isoprene and 2,3-Dimethyl-1,3-butadiene Rotamers," J. Mol. Struct.: THEOCHEM **187**, 69–82 (1989).

[6]A. Kraśnicki, K. Zbigniew, B. Drouin, and J. Pearson, "Terahertz Spectroscopy of isotopic Acrylonitrile," J. Mol. Spectrosc. **1006**, 20–27 (2011).

[7]J. Colmont, G. Wlodarczak, D. Priem, H. Müller, E. Tien, R. Richards, and M. Gerry, "Rotational Spectra of Selected Isotopic Species of Vinyl Cyanide: Molecular Structure and Quadrupole Hyperfine Structure," J. Mol. Spectrosc. **181**, 330–344 (1997).

[8]L. Kang and S. Novick, "The Microwave Spectra of the Weakly Bound Complex Between Carbon Monoxide and Cyanoacetylene, OC H-C≡C–C≡N," J. Mol. Spectrosc. **276-277**, 10–13 (2012).

[9]K. Matsumura, R. Suenram, F. Lovas, and T. Tanaka, "Fourier Transform Microwave Spectroscopy of Isotopically Substituted Diacetylenes: r_s-Structure, Quadrupole Coupling, and Anisotropic Nuclear Spin-Spin Interaction," J. Mol. Spectrosc. **240**, 120–126 (2006).

[10]G. Guirgis, C. Zheng, T. Gounev, and J. Durig, "Conformational Stability, Ab Initio Calculaitons, and r_o structural Parameters of 3-Methyl-1-butene and Dimethylvinylsilane," J. Mol. Struct. **651-653**, 771–780 (2003).

[11]A. Narten, "X-Ray Diffraction Study of Liquid Neopentane in the Temperature Range -17 to 150°C," J. Chem. Phys **70**, 299–304 (1979).

[12]M. Sugie, M. Kato, C. Matsumura, and H. Takeo, "Microwave Spectra and Molecular Structures of 1,2-Dichloroethane, 1,1-Dichloroethane and 1,1,1-Trichloroethane," J. Mol. Struct. **413-414**, 487–494 (1997).

[13]A. Almenningen, B. Andersen, and Trætteberg, "An Electron Diffraction Investigation of the Molecular Structure of Hexachloroethane in the Vapour Phase," Acta Chem. Scand. **18**, 603–611 (1964).

[14]R. Livingston and C. Ramachandra Rao, "The Molecular Structure of Pivalonitrile Electron Diffraction," J. Am. Chem. Soc. **81**, 3584–3586 (1958).

[15]T. Iijima, "An Electron-Diffraction Investigaton of the Molecular Structure of Toluene," Z. Naturforsch. **32a**, 1063–1064 (1977).

[16]F. Keidel and S. Bauer, "Structures of Toluene, Phenylsilane, and Diphenyldichlorosilane," J. Chem. Phys. **25**, 1218–1227 (1956).

[17]P. Scharfenberg, B. Rozsondai, and I. Hargittai, "Conformation and Structure of Ethylbenzene in the Vapour Phase," Z. Naturforsch. **35a**, 431–436 (1980).

[18]J. Cochran, K. Hagen, G. Paulen, Q. Shen, S. Tom, M. Trætteberg, and C. Wells, "On the Planarity of Styrene and its Derivatives: The Molecular Structures of Styrene and (Z)-β-Bromostyrene as Determined by Ab Initio Calculations and Gas-Phase Electron Diffraction," J. Mol. Spectrosc. **413-414**, 313–326 (1997).

[19]H. Rudolph, J. Demaison, and A. Császár, "Accurate Determination of the Deformation of the Benzene Ring Upon Substitution: Equilibrium Structures of Benzonitrile and Phenylacetylene," J. Chem. Phys. A **117**, 12969–12982 (2013).

[20]J. Casado, L. Nygaard, and G. Srensen, "Microwave Spectra of Isotopic Benzonitriles. Refined Molecular Structure of Benzonitrile," J. Mol. Struct. **8**, 211–224 (1970).

[21]L. Vilkov, N. Sadova, and S. Mochalov, "The Structure of Cumene and Phenylcyclobutane Molecules, as Studied by Means of Electron Diffraction Patterns," Dokl. Akad. Nauk SSSR **179**, 896–899 (1968).

[22]Q. Shen, C. Wells, M. Trætteberg, R. Bohn, A. Willis, and J. Knee, "Molecular Structure and Conformation of Cyclopropylbenzene as Determined by Ab Initio Molecular Orbital Calculations, Pulsed Jet Fourier Transform Microwave Spectroscopic, and Gas-Phase Electron Diffraction Investigations," J. Org. Chem. **66**, 5840–5845 (2001).

[23]A. Campanelli, F. Ramondo, A. Domenicano, and I. Hargittai, "Molecular Structure and Conformation of *tert*-Butylbenzene: A Concerted Study by Gas-Phase Electron Diffraction and Theoretical Calculations," J. Phys. Chem. **98**, 11046–11052 (1994).

[24]I. Karle and L. Brockway, "The Structures of Biphenyl, *o*-Terphenyl and Tetraphenylene," J. Am. Chem. Soc. **66**, 1974–1979 (1942).

[25]Q. Shen, "The Molecular Structure of 1,2-Diphenylethane as Determined by Gas-Phase Electron Diffraction," J. Mol. Struct. **471**, 57–61 (1998).

[26]M. Flamm-ter, H.-D. Beckhaus, K. Peters, H.-G. von Schnering, and C. Rüchardt, "2,3-Di-1-adamantyl-2,3-dimethylbutane; Long Bonds and Low Thermal Stability," Chem. Ber. **113**, 4665–4671 (1985).

[27]G. Kratt, H.-D. Beckhaus, H. Lindner, and C. Rüchardt, "Thermolabile Kohlenwasserstoffe, XX. Synthese, Struktur und Spannung Symmetrischer Tetraalkyldiarylethane," Chem. Ber. **116**, 3235–3263 (1983).

[28]D. Dougherty, C. Choi, G. Kaupp, A. Buda, J. Rudziński, and E. Osawa, "Effects of Substituents on the Length of Central C(sp^3)-C(sp^3) Bond in Anthracene Photodimers and Related Molecules," J. Chem. Soc. Perkin Trans. 2 **7**, 1063–1070 (1986).

[29]B. Slepetz and M. Kertesz, "Volume Change During Thermal [4+4] Cycloaddition of [2.2](9,10)Anthracenophane," J. Am. Chem. Soc. **135**, 13720–13727 (2013).

[30]E. Ehrenburg, "The Crystal Structure of Bi(anthracene-9,10-dimethylene) Photo-Isomer," Acta Cryst. **20**, 183–186 (1965).

[31]T. Takeda, H. Kawai, R. Herges, E. Mucke, Y. Sawai, K. Murakoshi, K. Fujiwara, and T. Suzuki, "Negligible Diradical Character for the Ultralong C–C Bond in 1,1,2,2-Tetraarylpyracene Derivatives at Room Temperature," Tetrahedron Lett. **59**, 3693–3697 (2009).

[32]K. Gunnelin, P. Glans, J.-E. Rubensson, C. Såthe, J. Nordgren, Y. Li, F. Gelḿukhanov, and H. Ågren, "Bond-Length-Dependent Core Hole Localization Observed in Simple Hydrocarbonds," Phys. Rev. Lett. **83**, 1315–1318 (1999).

[33]V. Amir-Ebrahimi and A. Choplin, "Microwave Spectrum of the ^{13}C-Ring-Monosubstituted Toluenes and Structure of Toluene," J. Mol. Spectrosc. **89**, 42–52 (1982).

[34]H. Okabe and V. Dibeler, "Photon Impact Studies of Cyanoacetylene and Acetonitrile in the Vacuum Ultraviolet. Heats of Formation of Ethynyl and Acetonitrile," J. Chem. Phys. **59**, 2430–2435 (1973).

[35]M. W. Chase and Jr., "Nist-janaf themochemical tables, fourth edition," J. Phys. Chem. Ref. Data Monograph **9**, 1–1951 (1998).

[36]J. Cioslowski, G. Liu, and D. Moncrieff, "Thermochemistry of Homolytic C–C, C-H, and C–Cl Bond Dissociations in Polychloroethanes: Benchmark Electronic Structure Calculations," J. Am. Chem. Soc. **119**, 11452–11457 (1997).

[37]R. Winiker, H.-D. Beckhaus, and C. Rüchardt, "Thermische Stabilität, Spannungsenthalpie und Struktur Symmetrisch Hexaalkylierter Ethane," Chem. Ber. **113**, 3456–3476 (1980).

[38]J. Martin, M. Fernandez, and J. Tortajada, "Application of Wiberg Indices to Geometry Optimization. C–C Distances," J. Mol. Struct. **175**, 203–208 (1988).

[39]L. Bartell and T. Boates, "Structure of the Strained Molecules Hexamethylethane and 1,1,2,2-Tetramethylethane by Gas-Phase Electron Diffraction," J. Mol. Struct. **32**, 379–392 (1976).

[40]P. R. Schreiner, L. V. Chernish, P. A. Gunchenko, E. Y. Tikhonchuk, H. Hausmann, M. Serafin, S. Schlecht, J. E. P. Dahl, R. M. K. Carlson, and A. A. Fokin, "Overcoming Lability of Extremely Long Alkane Carbon-Carbon Bonds Through Dispersion Forces," Nature **477**, 308–311 (2011).

[41]S. Grimme and P. R. Schreiner, "Steric Crowding Can Stabilize a Labile Molecule: Solving the Hexaphenylethane Riddle," Angew. Chem. Int. Ed. **50**, 12639–12642 (2011).

[42]B. Kahr, D. Van Engen, and K. Mislow, "Length of the Ethane Bond in Hexaphenylethane and its Derivatives," J. A. Chem. Soc. **108**, 8305–8307 (1986).

[43]Z. Szabo and I. Thege, "Some Empirical Correlations on Chemical Bonds," Acta Chem. Acad. Sci. Hung. **86**, 127–145 (1975).

[44]Y.-R. Luo, *Comprehensive Handbook of Chemical Bond Energies* (Taylor and Francis Group, NewYork, 2007).

[45]in *NIST Computational Chemistry Comparison and Benchmark Database, NIST Standard Ref-*

erence Database Number 101, Release 15b, edited by R. Johnson III (http://cccbdb.nist.gov/, 2011).

[46]H. Afeefy, J. Liebman, and S. Stein, in *NIST Computational Chemistry Comparison and Benchmark Database, NIST Standard Reference Database Number 69* (http:webbook.nist.gov/, 2001).

[47]D. R. Lide, *CRC Handbook of Chemistry and Physics, 74th ed.* (CRC, Boca Raton, FL, 1993).

[48]A. A. Zavitsas, "The Relation between Bond Lengths and Dissociation Energies of Carbon-Carbon Bonds," J. Phys. Chem. A **107**, 897–898 (2003).

[49]D. Griller, J. Kanabus-Kaminska, and A. Maccoll, "Bond Dissociation Energies for Common Organic Compounds," J. Mol. Struct.: THEOCHEM **40**, 125–131 (1988).

[50]M. P. Johansson and M. Swart, "Intramolecular halogen–halogen bonds?" Phys. Chem. Chem. Phys. **15**, 11543–11553 (2013).
